# Interpretable Machine Learning Reveals Dissimilarities Between Subtypes of Autism Spectrum Disorder

**DOI:** 10.3389/fgene.2021.618277

**Published:** 2021-02-25

**Authors:** Mateusz Garbulowski, Karolina Smolinska, Klev Diamanti, Gang Pan, Khurram Maqbool, Lars Feuk, Jan Komorowski

**Affiliations:** ^1^Science for Life Laboratory, Department of Cell and Molecular Biology, Uppsala University, Uppsala, Sweden; ^2^Science for Life Laboratory, Department of Immunology, Genetics and Pathology, Uppsala University, Uppsala, Sweden; ^3^Swedish Collegium for Advanced Study, Uppsala, Sweden; ^4^Institute of Computer Science, Polish Academy of Sciences, Warsaw, Poland; ^5^Washington National Primate Research Center, Seattle, WA, United States

**Keywords:** autism spectrum disorder, interpretable machine learning, transcriptomics, rule-based classification, autism spectrum disorder subtypes, data integration, gene expression

## Abstract

Autism spectrum disorder (ASD) is a heterogeneous neuropsychiatric disorder with a complex genetic background. Analysis of altered molecular processes in ASD patients requires linear and nonlinear methods that provide interpretable solutions. Interpretable machine learning provides legible models that allow explaining biological mechanisms and support analysis of clinical subgroups. In this work, we investigated several case-control studies of gene expression measurements of ASD individuals. We constructed a rule-based learning model from three independent datasets that we further visualized as a nonlinear gene-gene co-predictive network. To find dissimilarities between ASD subtypes, we scrutinized a topological structure of the network and estimated a centrality distance. Our analysis revealed that autism is the most severe subtype of ASD, while pervasive developmental disorder-not otherwise specified and Asperger syndrome are closely related and milder ASD subtypes. Furthermore, we analyzed the most important ASD-related features that were described in terms of gene co-predictors. Among others, we found a strong co-predictive mechanism between *EMC4* and *TMEM30A*, which may suggest a co-regulation between these genes. The present study demonstrates the potential of applying interpretable machine learning in bioinformatics analyses. Although the proposed methodology was designed for transcriptomics data, it can be applied to other omics disciplines.

## Introduction

Autism spectrum disorder (ASD) is a neurodevelopmental disorder that has been extensively studied over the past decades (Cox et al., 1999; Marshall et al., 2008; Lord et al., 2018; Ozonoff and Iosif, 2019). The highly heterogeneous neurodevelopmental changes in ASD commonly lead to challenges in social interactions and communication and contribute to restricted and repetitive behaviors (Sharma et al., 2018). Diagnosis of ASD and its severity is typically performed with interviews of the proband and their family (Sharma et al., 2018). Epidemiological studies have shown that ASD has high heritability, but the genetic etiology is complex and heterogeneous (Tick et al., 2016; Feliciano et al., 2019). Transcriptomics studies have found that gene expression changes in blood of ASD subjects are linked to specific risk factors that may support the diagnosis (Gregg et al., 2008; Xiong et al., 2019).

Despite the fact that ASD is associated with the central nervous system, blood is frequently used for ASD research (Ansel et al., 2017). Although brain tissue samples are more relevant to explore ASD biomarkers, it is difficult to obtain samples from living subjects; thus, they are usually extracted postmortem. Recent studies (Fiorentino et al., 2016; Kealy et al., 2018) have shown that the blood-brain barrier (BBB) is altered in patients with psychiatric disorders, including ASD. Furthermore, one of the major effects of BBB dysfunctions is changes in the immune system. It has been shown that the gene expression profile of NK cells was altered in peripheral blood of children with ASD (Enstrom et al., 2009). Another transcriptomics data analysis of ASD subjects demonstrated similarities in functional enrichment between brain and blood (He et al., 2019). The study revealed significant enrichment in terms of immune response, mitochondrion-related functions, and oxidative phosphorylation. In addition, various advantages of using blood to study ASD individuals have been well-described by Ansel et al. (2017).

Clinicians have discerned several subtypes of ASD that share common behaviors (Witwer and Lecavalier, 2008). Commonly diagnosed ASD subtypes are (i) pervasive developmental disorder-not otherwise specified (PDD-NOS), (ii) Asperger syndrome (AS), and (iii) autism. Earlier studies have investigated similarities among ASD subtypes. In Walker et al. (2004), comparison of autistic symptoms revealed that PDD-NOS is less severe than autism and AS. In addition, AS and PDD-NOS were also shown to be closely related in terms of social functioning level. A comparative study by Li et al. (2019) tested genetic components and gene expression patterns of the ASD subtypes. These studies have shown that autism and PDD-NOS share broad similarity, while AS exhibits distinct patterns.

In recent years, different machine learning approaches have been successfully applied to mine knowledge from various types of omics (Oh et al., 2017; Chand et al., 2020; Maros et al., 2020). These analyses have assisted in biomarker identification and better understanding of the underlying biological processes for various inherited disorders. As numerous studies have produced large datasets, there is a need to efficiently merge information from multiple sources to increase its statistical power and interpretability (Lagani et al., 2016; Dong and Rekatsinas, 2018; Fajarda et al., 2020). In bioinformatics, machine learning is a powerful technique for data integration and analysis (Li et al., 2018). However, most of the commonly used algorithms are black-box approaches that frequently lead to poor interpretability of the classifier (Rudin and Radin, 2019). Various studies (Ali et al., 2018; Orange et al., 2018; Gao et al., 2019; Matsui et al., 2020; Sinkala et al., 2020) performed a machine learning analysis on disease subtype classification by constructing models that its internal structure is difficult to explain. Herein, we proposed the utilization of interpretable machine learning (IML; Molnar, 2020) to perform an integrative analysis on multiple transcriptomics datasets. IML algorithms allow for visibility of the internal decisions made by the system. In this work, interpretability is intrinsically determined by a set of IF-THEN rules that constitute a rule-based model. More importantly, such IML models can be visualized in various graphical forms (Bornelöv et al., 2012; Anyango, 2016). Therefore, we focused on graphic representations of IML models as a co-predictive undirected network that allowed us to explore dissimilarities among clinical subgroups.

**Figure 1:**
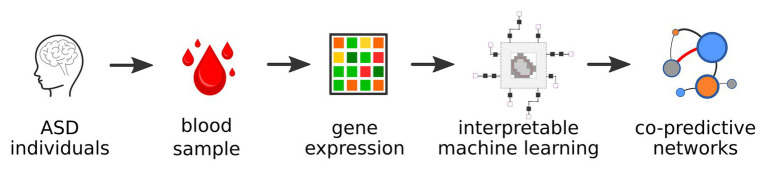
General workflow of the analysis.

In this study, we examined dissimilarities of ASD subtypes identified from IML modeling using three transcriptomics cohorts. Herein, we assumed interpretability of the IML modeling over its performance. We showed that IML modeling is capable of single-view integrative analysis of predefined ASD subtypes (Figure 1). Visualization of the ASD-control studies in a single network revealed a strong co-predictive mechanism between *EMC4* and *TMEM30A* and other mechanisms. To analyze ASD subtypes, we measured the distance among subnetworks representing ASD subtypes and established dissimilarities between autism, AS, PDD-NOS, and control. Based on the network structure and node connection parameters, we found that AS is a milder form of ASD and autism is the most severe form of ASD. Finally, we performed functional profiling of the genes used for IML modeling to examine functional information in various databases. The results from this study showed that rule-based IML can be applied on an integrative analysis and to estimate co-predictors of ASD. Furthermore, based on co-predictive genes, rule-based modeling can be used for describing dissimilarities between ASD subtypes and control.

## Materials and Methods

### Overview of the Workflow

**Figure 2:**
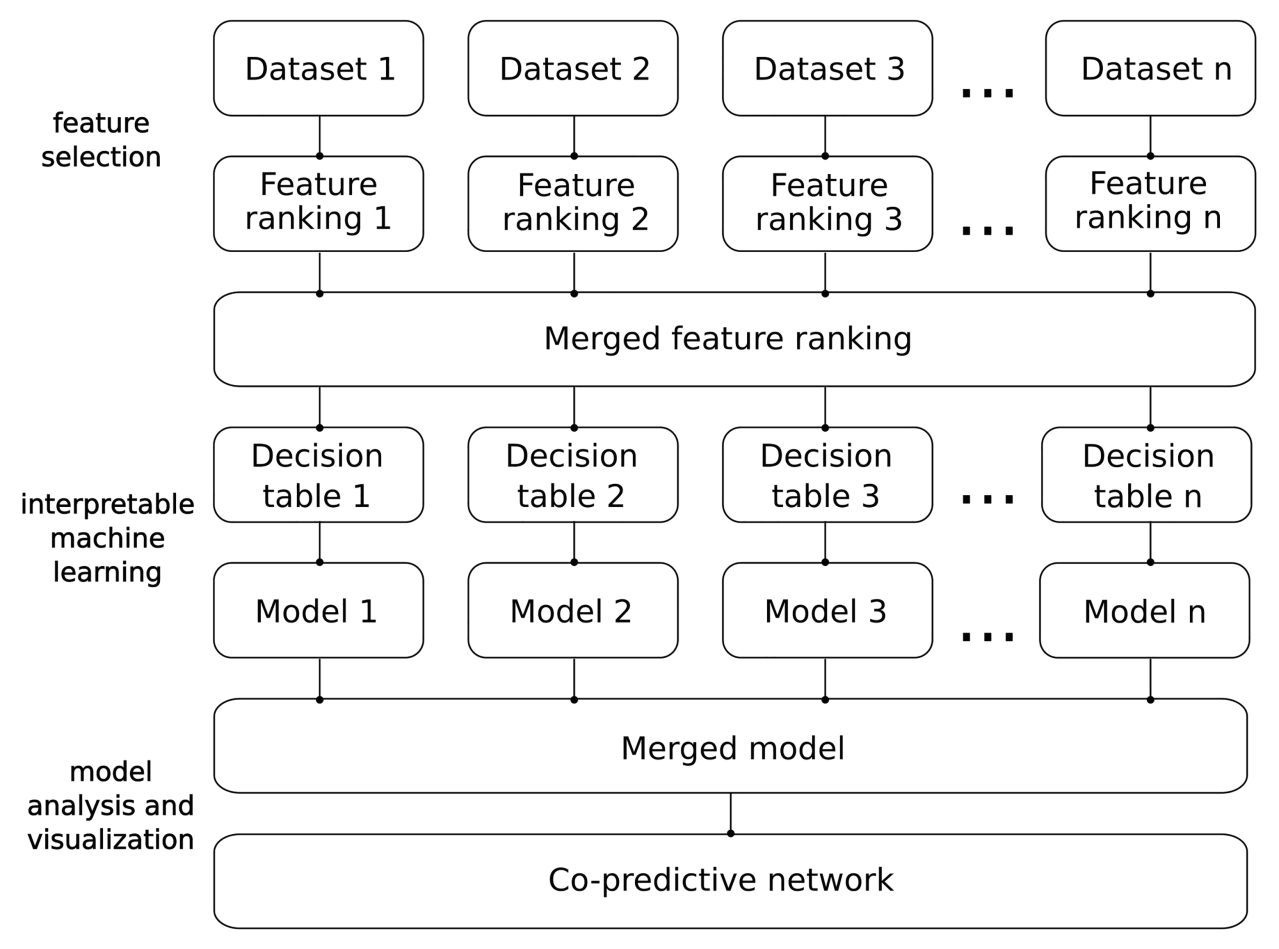
From data to network. Detailed pipeline of the analysis.

Our methodology was designed to construct and analyze unbiased IML models. A schematic overview of the pipeline we employed to construct and visualize the IML model is shown in Figure 2. This methodology can be applied to data from any omics discipline that can be transformed into a decision table, e.g., RNA-seq, DNA methylation, or mass spectrometry proteomics. Details of the analysis are described in the subsections below.

### Datasets Description and Preprocessing

We collected three datasets of case-control studies of ASD that we named DS1 (Kong et al., 2012), DS2 (Kong et al., 2012), and DS3 (Alter et al., 2011). These three datasets contained gene expression levels measured with Affymetrix arrays. Datasets were selected based on sampling tissue being peripheral blood, contain a relatively high number of samples for IML modeling (more than 30 per decision class) and are publicly available in the Gene Expression Omnibus (GEO) repository. In total, 431 samples were collected and analyzed (Table 1).

**Table 1:** Overview and accession numbers of the datasets used in the analysis.

Dataset	Author	GEO series	Affymetrix array	No ASDs	No controls	No genes	Tissue	ASD subtypes
DS1	Kong et al., 2012	GSE18123	Human Genome U133 Plus 2.0	66	33	54,676	Peripheral blood	PDD-NOS AS autism
DS2	Kong et al., 2012	GSE18123	Human Gene 1.0 ST	104	82	33,298	Peripheral blood	PDD-NOS AS autism
DS3	Alter et al., 2011	GSE25507	Human Genome U133 Plus 2.0	82	64	54,676	Peripheral blood lymphocytes	Autism
DS4	Gregg et al., 2008	GSE6575	Human Genome U133 Plus 2.0	35	12	54,676	Peripheral blood	Autism

Raw gene expression data were imported with the recent versions of R packages *affy* (DS1 and DS3) and *oligo* (DS2; Gautier et al., 2004; Carvalho and Irizarry, 2010). We performed Robust Multi-array Average (RMA) normalization and background correction on the datasets. Furthermore, each of the datasets was investigated for known and latent batch effects (Supplementary Figure S1). We performed a principal component analysis (PCA) and inspected the impact of clinical variables such as age, sex, or other unknown sources. We found that DS3 may be affected by the age disproportionality of the subjects and the data was corrected for this factor using *ComBat* (Leek et al., 2012). To detect latent batch effects, we estimated surrogate variables. As a consequence, DS1 and DS2 were corrected for unknown biases with 2 and 17 significant surrogate variables, respectively. To adjust for detected biases, we used the *sva* and *limma* packages (Smyth, 2005; Leek et al., 2012). To evaluate highly ranked co-prediction mechanism in ASD, we introduced DS4 (Gregg et al., 2008), which was preprocessed using the same approaches as for other datasets (Supplementary Figure S2). DS4 was excluded from IML modeling due to small sample size of the control class (Table 1). Thus, control samples from DS4 were not used for validation of co-predictors. In addition, one of the control samples from DS4 was distinguished as an outlier (Supplementary Figures S2A,C) and removed.

### Feature Selection

Microarray gene expression data measure the expression levels of up to about 60,000 genes. However, the considerably smaller number of samples introduces an ill-defined problem that we solved employing feature selection. To reduce the high dimensionality of the data, we performed Monte Carlo feature selection (MCFS) that ranks the features based on their estimated relative importance (RI) from decision trees (Dramiński et al., 2008). To generate the ranking of the most important genes and exclude redundant signals, we used the R package *rmcfs* (Dramiński and Koronacki, 2018). In order to avoid overfitting, we used the results from *rmcfs* of all datasets in a cross-validation (CV) manner (Krawczuk and Łukaszuk, 2016). To select the top features estimated by MCFS, we chose a critical angle thresholding method (Supplementary Figure S3). Other methods for the selection of the RI threshold included mean, k-means, and the permutation-based approach. The permutation-based method is the default for *rmcfs*; however, for DS2 and DS3 it returned only one feature, making it non-feasible to perform learning (Supplementary Figure S3A). In contrast, mean and k-means chose very relaxed thresholds which resulted in a very large number of features potentially introducing noise to the learning process (Supplementary Figure S3B). On the other hand, critical angle showed consistency by selecting a similar number of features across datasets (Supplementary Figure S3A). Finally, we constructed a merged feature ranking (FR) of the most relevant features sorted by their RI.

#### FR Adjustment

To correct for inconsistency between feature selection and classification techniques, we adjusted the number of the most relevant features given by the MCFS. To account for the diversity between *rmcfs* and *R.ROSETTA* algorithms, we introduced correction of the threshold of the most important features. To find the new number of features, we estimated accuracy and area under the ROC curve (AUC) for several models using diverse numbers of top features (Supplementary Figure S4). By taking the original FRs from *rmcfs*, we iteratively added features, one by one, with respect to their decreasing RI. The process started with the number of features given by a critical angle threshold from *rmcfs* and proceeded until the ranking reached 50 features. Each FR was used to construct a decision table and perform rule-based classification. Finally, to avoid overestimation we chose the number of top features from the model with the nearest local maximum of the highest model quality (Supplementary Figure S4). As a result of FR adjusting, the final models gained slightly more features relevant from the rule-based modeling perspective. The final FR consisted of 50 genes.

### Rule-Based Classification

In this work, we based the IML approach on the rough set theory (Pawlak, 1982; Pawlak and Skowron, 2007). In the rough set theory, the data universe consists of examples, which may also be called objects or samples, and variables also called attributes or features. By marking the last attribute as a decision class, the structure called a decision table is constructed (Pawlak, 2002). The rough set-based approach assumes that examples with exactly the same information are indiscernible (Skowron and Dutta, 2018). In this study, objects are represented by the ASD samples and attributes by the set of the most informative genes selected from adjusted FRs. Importantly, rough set-based models estimate reducts that determine minimal sets of attributes (Komorowski, 1999). Moreover, reducts are the main components for estimating rules that represent a co-predictive mechanism of features. In this type of IML, the set of rules constitutes the model legibility. A single rule is represented as an IF-THEN formula in natural language. Specifically, IF certain conditions are fulfilled THEN a prediction is made. Rules are often accredited by the fundamental measures of support, coverage and accuracy (Tsumoto, 2002; Molnar, 2020). The rule support (RS) is the number of samples that fulfill the rule conditions or prediction. We discern between the left-hand site rule support (RS_LHS_) that corresponds to the IF-part of the rule (conditions) and the right-hand site rule support (RS_RHS_) that corresponds to the THEN-part of the rule (prediction). The rule coverage (RC) is decision class-specific fraction of samples that match the rule. For example, if 30 out of 100 samples fulfill the THEN-part of the rule then RC_RHS_ of such rule is 0.3. The RC is defined as support divided by the total number of samples for a given decision class that is represented as:

$$\mathrm{RC(rule)}=\frac{\mathrm{RS(rule)}}{n_{d}}$$

where RS is the LHS or RHS support of the rule and n_d_ is the total number of samples for a decision class.

The rule accuracy (RA) describes the predictive power of the rule based on RS. For example, if the IF-part of the rule corresponds to 10 samples and THEN-part corresponds to 9 then RA is 0.9. Specifically, RA is calculated as:

$$\mathrm{RA(rule)}=\frac{{\mathrm{RS}}_{\mathrm{RHS}}\mathrm{(rule)}}{{\mathrm{RS}}_{\mathrm{LHS}}\mathrm{(rule)}}$$

where RS_RHS_ is the right-hand site support and RS_LHS_ is the left-hand site support for a rule.

Based on these basic measurements, other statistical values can be estimated, such as rule value of *p* (Garbulowski et al., 2020). Additionally, the length of a rule is also an important factor. If the rule consists of two or more conditions, then a co-predictive mechanism occurs for these features. Herein, we used rule-statistic measures for pruning and assessing the IML model quality.

To construct legible classifiers, we used a rule-based framework that receives a decision table as input and generates IML model as output. The modeling was performed with R package *R.ROSETTA*, which is a wrapper of the *ROSETTA* system (Øhrn and Komorowski, 1997; Garbulowski et al., 2020). The IML modeling was performed using 10-fold CV and the standard voting method. The datasets were discretized using equal frequency binning with three levels on the training set, and subsequently the cuts were applied to the test set. The Johnson reduction method was used to generate the reducts and rules for the models. Furthermore, we used undersampling to account for uneven distribution of samples in each class across datasets. After balancing the data, *R.ROSETTA* recalculated all the statistical values for rules according to the information from nonsampled examples. The result of recalculation allowed finding the exact samples that correspond to rules, known as support sets. These were further used to link rules with their clinical subgroups.

The final rule-based models were constructed from the merged FR sets. However, due to differences between microarray platforms (Table 1) the overlap between sets of genes was incomplete. Thus, two genes were not used for classification in DS1 and DS3, and nine genes were not used for classification in DS2 (Table 2).

**Table 2:** Characteristics and results of IML models built on the original and merged FRs.

FR	Model characteristic	DS1	DS2	DS3
Original	Number of features	19	13	18
	Number of rules	358	790	565
	Accuracy	78%	75%	69%
	AUC	0.83	0.80	0.78
Merged	Number of features	48	41	48
	Number of rules	367	481	623
	Accuracy	75%	70%	67%
	AUC	0.82	0.75	0.72

### Merging Datasets

The main advantage of IML-based analysis is to obtain legible models that can be easily analyzed. Data integration is an important task that unifies different datasets and increases the statistical power of the analysis (Lenzerini, 2002). In this study, we proposed two merging steps for (i) the most important features and (ii) IML models (Figure 2). Respective adjusted FRs were merged into a single FR that consisted of all the important features selected by MCFS. As datasets were produced with different microarray platforms, we compared the positions of probes and remapped probe IDs across platforms. The positions were compared using the R package *GenomicRanges* (Lawrence et al., 2013) and the UCSC Genome Browser (Kent et al., 2002). To merge IML models, we aggregated rules from all models. For rules that occurred multiple times its RS and support sets were summed up. In consequence, RA, RC, values of *p*, and other statistics measures of rules were normalized to merged cohorts and recalculated. The result of the pipeline (Figure 2) that included these merging steps is a single model built from multiple datasets. The model was further used to create the rule-based networks.

### Co-predictive Network

In this step, we visualized the merged IML model of ASD as a rule-based network using the *VisuNet* R package (Anyango, 2016; Smolinska, 2021). The network displays co-predictive mechanisms of features that are defined as nodes and edges. Each node is described by the RS or RC and its connection parameter. The filtration methods in *VisuNet* help show the most relevant elements of the network. Furthermore, *VisuNet* allows presenting gene expression levels with predefined colors of nodes. Thus, it assists toward better interpretation of the IML models. Herein, such a graphic representation was used to display co-predictive genes for the merged ASD model.

The two main points of interest while analyzing rule-based networks are hubs and large nodes. A hub is a node that connects to multiple other nodes, and it is marked with a thick blue border. The interpretation of a hub may suggest a feature that frequently participates in co-prediction, in other words, a feature that is a good predictor in combination with many other features. Large nodes represent features that are supported by many samples.

### Network Comparison

#### Network Structure

To analyze the network structure, we investigated the connection parameter. For an edge that connects two nodes, a connection value is defined as:

$$\mathrm{connection}\left(\mathrm{x,y}\right)=\underset{\mathrm{rule}\in R\left(\mathrm{x,y}\right)}{\sum }\mathrm{RS}\left(\mathrm{rule}\right)\times \mathrm{RA}\left(\mathrm{rule}\right)$$

where *x* and *y* are features with their discretization levels of the rule, RS is the rule support, and RA is the rule accuracy.

The connection value for the edge is unity-based normalized. For nodes, the connection is defined as the sum of all connections between the given node and all other connected nodes. Herein, the connection can be interpreted as a nonlinear association between two or more genes. To examine the contribution of genes for discerning among ASD subtypes, we used node connection values for clustering. As the connection values were not normally distributed, we performed clustering with Kendall correlation metrics (Abdi, 2007). We scaled all connection values between 0 and 1, and then, for clustering, we selected all the nodes that describe genes on their discrete expression levels.

#### In-Between-Network Distance

To characterize dissimilarities between clinical subgroups, we linked rules from the merged model with various clinical variables such as ASD subtypes, age, and sex (Supplementary Table S1). Using the *R.ROSETTA* package, we recalculated the model and extracted support sets for each rule that were further translated to the particular clinical subgroup. Dissimilarities between subgroups were based on estimating the centrality distance for the networks (Borgatti and Everett, 2006; Roy et al., 2014). In particular, centrality betweenness distance is estimated based on the shortest path between two given nodes. We tested several approaches of estimating in-between graph distance with the *NetworkDistance* R package (You, 2020). To validate our findings, we performed a permutation test by shuffling the decision of the rules for each network 500 times. Additionally, as the proportion of the recalculated rules among the decision classes was imbalanced, we iteratively sampled equal numbers of rules 20 times and averaged the distance for balanced networks. We assumed that the distance for random networks cannot be greater than the distance for original networks; thus, we estimated left-tailed values of *p*.

### Functional Profiling

The functional profiling of the genes from merged FR was performed with the web-based toolset *g:Profiler* that performs enrichment analysis (Reimand et al., 2007). We used a large collection of sources for sets of gene including gene ontology (GO) for molecular function, cellular component and biological processes, Kyoto Encyclopedia of Genes and Genomes (KEGG), Reactome, TRANSFAC, miRTarBase, Human Protein Atlas, CORUM protein complexes, human phenotype ontology, and WikiPathways. The functional enrichment analysis was performed using *g:GOSt* with the false discovery rate (FDR) set to 5%. The profiling could be executed for the set of 40 out of 50 genes that were identified by the tool (Supplementary Table S2).

Additionally, we evaluated the results of the functional profiling for specific ASD subtypes. We selected top co-predictors from the network by estimating thresholds using the k-means method from R package *mmand* (Clayden et al., 2020). Specifically, thresholds were estimated based on the node connection values from the rule-based networks. The list of estimated top co-predictors can be found in Supplementary Table S3. In the next step, we calculated the fraction of genes that correspond to selected terms. For example, if 5 out of 10 top co-predictors intersected with genes included in a given term, the fraction is 0.5. Terms were selected according to ASD-associated terms found in literature. These include alterations related to the immune system (Ormstad et al., 2018), calcium (Guan et al., 2020), metabolism (Shmais et al., 2012; Frye et al., 2013), mitochondrion (Rossignol and Frye, 2012), metal ions (Hagmeyer et al., 2015), and membrane (Kitagishi et al., 2015).

## Results

### IML Modeling and Visualization

Following the pipeline (Figure 2), we first applied MCFS and created a merged FR from the original FRs of the respective datasets. As mentioned in the FR adjustment subsection, the thresholds for selecting features were adjusted in order to increase the number of genes associated with the rule-based classification. While merging, we found no overlapping genes between FRs. Next, we compared the difference in model quality introduced by merging FRs from the individual datasets. Models that were built on FRs computed from the same dataset resulted in a reasonable quality (Table 2). The average accuracy was 74%, and the average AUC was 0.80. After introducing the merged FR for modeling, we observed a moderate drop in quality. The latter suggests a reduction on the bias introduced by employing features applied on the IML of the same dataset. Models for DS1 and DS3 based on the set of merged FRs resulted in an increase in the number of rules as compared to the ones that were based on the dataset-specific FR, while the opposite occurred for DS2 suggesting overfitting in the original model (Table 2). Additionally, we observed that DS3 resulted in the lowest quality across models. It suggests that the quality of this dataset may be lower than DS1 and DS2. Importantly, DS3 consisted only of autistic male subjects; thus, the data variability may be lower than for DS1 and DS2. Despite the fact that each dataset obtained a unique set of the most important features, contribution of all features from merged FRs has been observed in IML modeling and further in networks (Supplementary Figure S5). The weakest contribution has been observed from DS3 for ASD samples (Supplementary Figure S5A). For control, genes contributed equally in modeling (Supplementary Figure S5B).

**Figure 3:**
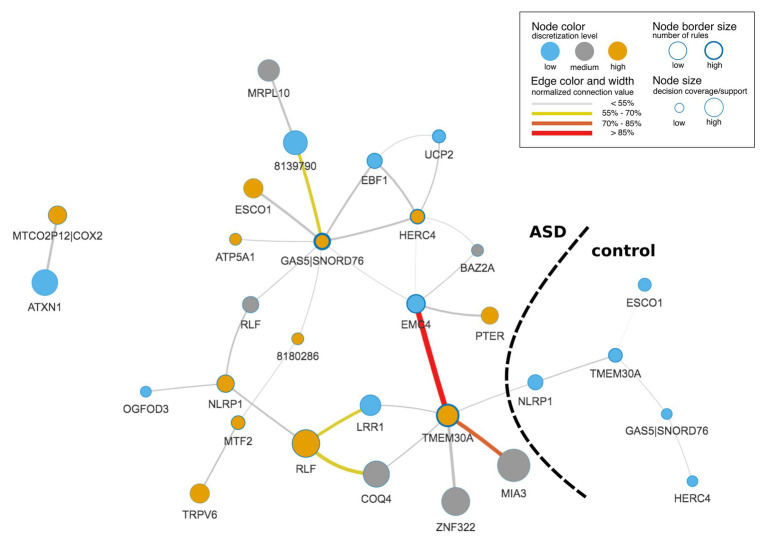
Rule-based network displaying top co-predictions of an IML model of case-control studies for autism spectrum disorder (ASD). The network was built with significant rules (false Discovery Rate, FDR-corrected *p* ≤ 0.05) for both classes together and illustrates top 5% of the 30 most strongly connected nodes. Edge and node connection values represent strength of the co-predictors in the classification procedure. Black dashed line divides network into subnetworks that correspond to ASD and control classes.

The final IML model contained 1,448 rules (693 for ASD and 755 for control) that covered all samples and important genes from DS1, DS2, and DS3. A graphic representation of the IML model demonstrated the co-predictive mechanisms of genes for different expression levels toward ASD (Figure 3). Interestingly, a few hub nodes were distinguished on the network that corresponded to *EMC4*, *GAS5|SNORD76*, *HERC4*, and *TMEM30A*. This suggested that these genes were highly relevant co-predictors that discern between ASD and control subjects. Further analysis on the edges of the network highlighted a strong connection between low expression of *EMC4* and high expression of *TMEM30A* (Figure 3) that we explored in detail below.

### Co-predictive Mechanisms

**Figure 4:**
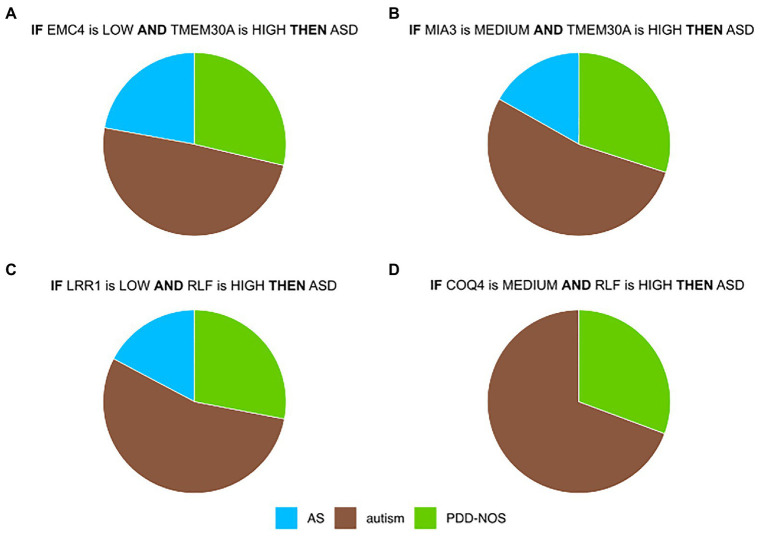
Top co-predictors derived from the ASD-control network. **(A)** Distributions of ASD subtypes of selected co-predictive mechanisms for **(A)** *EMC4*-*TMEM30A* **(B)** *MIA3*-*TMEM30A* **(C)** *LRR1*-*RLF*, and **(D)** *COQ4*-*RLF*. Values were normalized to the total amount of objects for each ASD subtype.

Most prominent is a co-regulation between *EMC4* and *TMEM30A* (Figure 3, Supplementary Figure S6). In control samples of DS1 and DS2, their correlation was close to 0 (Supplementary Figures S6A,B), while they were co-regulated in ASD samples for DS1 and DS2 (Supplementary Figures S6D,E). In contrast, we did not observe significant differences in DS3 (Supplementary Figures S6C,F). It may be due to a lower quality of the IML model built from DS3. This *in silico*-identified co-predictive mechanism suggested that this dependency may consist a co-expression mechanism. Additionally, we performed a statistical analysis on the particular genes in order to validate their expression changes between ASD and control (Supplementary Figures S6G–I). We observed that expression of both genes was significantly changed in DS1 (Supplementary Figure S6G) and DS2 (Supplementary Figure S6H). Furthermore, the respective studies, DS1 and DS2, also found *TMEM30A* as a differentially expressed gene that was further confirmed with qRT-PCR (Kong et al., 2012). We also evaluated other co-predictive mechanisms that were visible in the network (Figure 3). These include *MIA3*-*TMEM30A*, *LRR1*-*RLF*, and *RLF*-*COQ4* (Supplementary Figures S7–S9). These co-predictions were detected for DS1 and DS2 and validated with DS4 (Supplementary Figure S10). To investigate ASD subtypes for these co-predictive mechanisms, we evaluated their support sets and its percentage distribution normalized to the total amount of ASD subtypes (Figure 4).

### Dissimilarities of ASD Subtypes

We assumed that the concept of rule-based networks follows the same principles as any other undirected network. Thus, node‐ or edge-oriented properties can be analyzed and the distance for networks can be estimated (Entringer et al., 1976). Previous studies have shown that measuring the distance between networks can assist on bioinformatics analysis (Notebaart et al., 2008;Chen et al., 2018). Since a rule-based network is a more descriptive way of evaluating IML models, we used its topological structure to perform an analysis on ASD clinical subgroups. To provide the dissimilarity values between the subgroups, we measured centrality distances for the networks and performed a permutation test (Borgatti and Everett, 2006; van Borkulo et al., 2015). Among other methods (You, 2020), we found that only betweenness centrality distance resulted in significant values of *p* from permutation tests.

**Figure 5:**
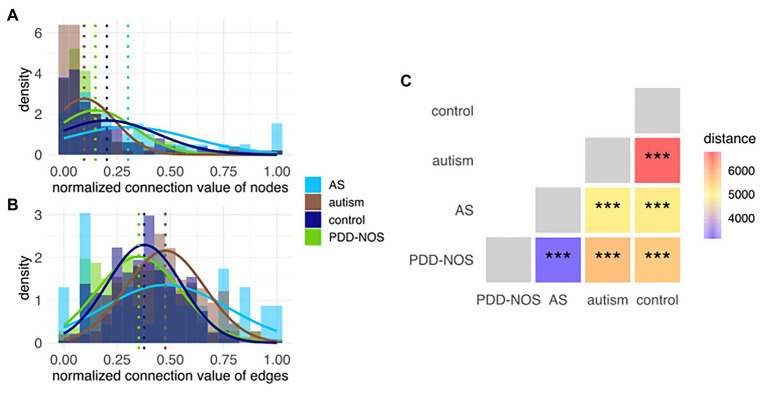
Histogram of the connection values for **(A)** nodes and **(B)** edges. Each histogram represents subnetworks that correspond to the particular ASD subtype and control. Modeled distributions were marked for each subgroup. Dotted lines represent the average values for each distribution. **(C)** In-between-network dissimilarity estimated as a centrality betweenness distance. Values of *p* were marked as ns (*p* > 0.05), ^*^(*p* ≤ 0.05), ^**^(*p* ≤ 0.01), and ^***^(*p* ≤ 0.001).

We first recalculated the model according to the clinical subgroups (cf. section Materials and Methods) that allowed creating clinical subgroup-specific subnetworks. To validate if the network structure could be used for finding differences between subgroups, we used node and edge connection values, which represent the connection power. Modeled distributions showed varying differences between subgroups; therefore, this confirmed that the network structure was capable of discerning subgroups (Figures 5A,B). To obtain an intuitive measure of these differences, we estimated the betweenness centrality among pairs of subnetworks and confirmed the robustness of the distances using permuted sets of networks (Figure 5C, Supplementary Figure S11).

Autism and control were the most dissimilar subgroups based on their subnetwork distances (Figure 5C). Comparison among all pairs of phenotypes sorted ASD subtypes in a decreasing fashion based on the distance from control, that is, autism, PDD-NOS, and AS (Figure 5C). The latter suggested that autism was the most severe form of ASD, PDD-NOS is milder than autism, while AS is the mildest form of ASD. This result is consistent with previous studies (Walker et al., 2004). Furthermore, network-based distance analysis suggested that PDD-NOS and AS are closely related subtypes (Figure 5C). In addition, we investigated age and sex subgroups; however, the permutation test resulted in nonsignificant (*p* > 0.05) associations (Supplementary Figures S12–S15).

**Figure 6:**
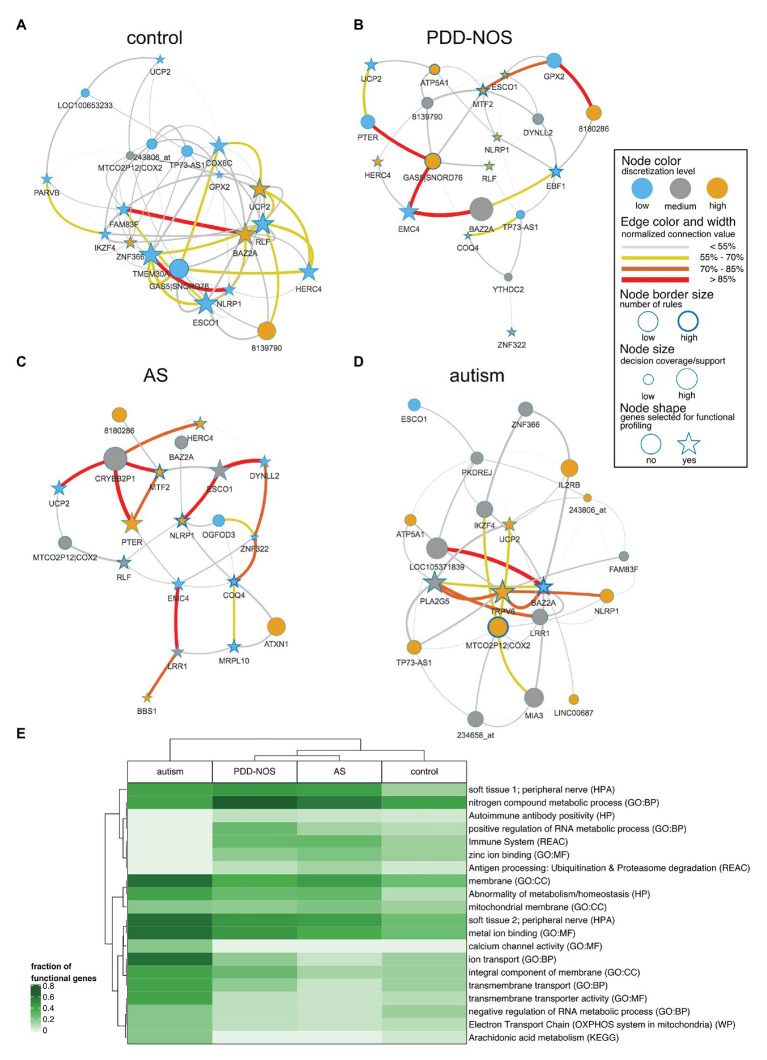
Network representation for particular ASD subtypes and control. To simplify the interpretation, subnetworks were pruned for the most strongly connected 20 nodes. Stars represent the strongest nodes estimated with the k-means algorithm based on node connection. A figure displays subnetworks for **(A)** control, **(B)** AS, **(C)**, PDD-NOS, and **(D)** autism. **(E)** Heat map of the fraction of genes included in functional terms related to ASD. Terms were presented for following databases: gene ontology – biological process (GO:BP), gene ontology – cellular component (GO:CC), gene ontology – molecular function (GO:MF), Human Phenotype Ontology (HP), Human Protein Atlas (HPA), Kyoto Encyclopedia of Genes and Genomes (KEGG), Reactome (REAC) and WikiPathways (WP).

We pruned subnetworks for various phenotypes to identify genes that discern between ASD subtypes and control (Figures 6A–D). We observed genes that were shared across the subgroups such as *UCP2* (control, PDD-NOS, autism, and AS) or highly expressed MTF2 (PDD-NOS and AS). We also observed unique genes for specific subgroups such as *TRPV6* for autism or *ATXN1* for AS. Furthermore, we identified ASD subgroup-specific hubs, for example, *GAS5*|*SNORD76* and *MTF2* for PDD-NOS or *BAZ2A* and *MTCO2P12|COX2* for autism. To evaluate topological similarities of networks, centrality betweenness distance finds the shortest path between the given pair of nodes. For example, interactions for *MTF2*-*ESCO1*-*DYNLL2* (Figure 6C) and *MTF2*-*DYNLL2* (Figure 6B) shall be considered as similar structures between PDD-NOS and AS.

**Figure 7:**
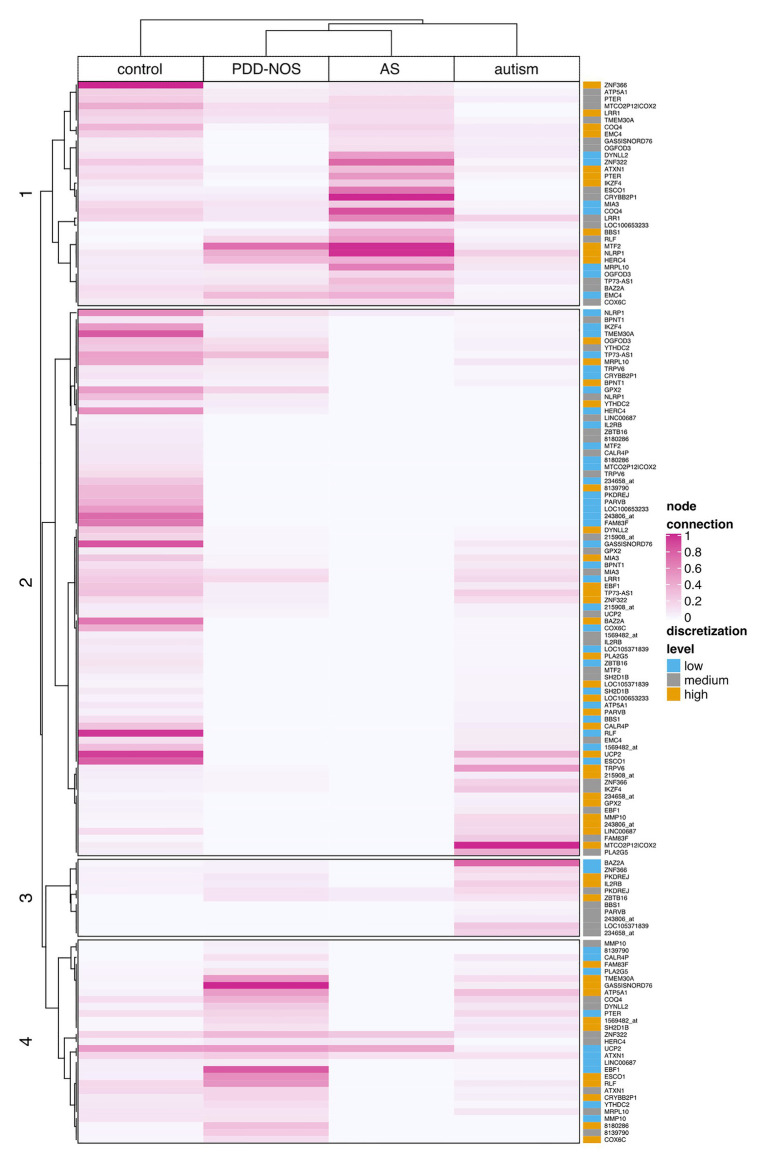
Clustering of the node connection values based on Kendal correlation. A heat map displays the result of clustering for particular ASD subtypes and control. Values were scaled between 0 and 1 column-wise. The rightmost panel shows discretization levels for gene expression values. A dark pink color indicates nodes with many connections (hubs), while light pink or white indicates weak connection or lack of the connection, respectively.

To examine all genes that discerned among subgroups, we clustered ASD subtypes by node connection values. Patterns of co-predictive mechanisms grouped ASD subtypes and confirmed our previous findings (Figure 7). The control group was well-separated from the ASD subtypes. Furthermore, PDD-NOS and AS were clustered together, which again suggested a degree of similarity between subtypes. We observed that genes (rows) were divided into four main branches that marked different gene groups, named 1, 2, 3, and 4 (Figure 7). Groups of genes in these branches could be described as follows: (1) majority of highly connected genes for AS and stronger connections shared between PDD-NOS and AS; (2) majority of highly connected and lowly expressed genes for control; (3) majority of highly connected genes for autism and lowly connected genes for control, PDD-NOS and AS; and (4) majority of highly connected genes for PDD-NOS. Specifically, genes from group 4 indicate a few shared patterns between control, PDD-NOS, and AS, while genes from group 2 show no connections for AS.

### Functional Profiling

To evaluate functions of the genes included for IML modeling, we performed functional profiling using *g:Profiler* for GO terms and biological pathways. From the GO annotations for molecular functions, we observed enrichment for binding molecules, such as metals, cations, ions, DNA nucleic acids, transcription factors, and zinc (FDR-corrected *p* < 0.01). Additionally, enriched GO biological processes highlight transmembrane transport and metabolic processes. The GO enrichment analysis for cellular components led to general terms related to membranes and cellular structures. From the Human Protein Atlas, we observed that *LRR1*, *ATXN1*, *TMEM30A*, *YTHDC2*, *EMC4*, *BPNT1*, *ZNF322*, *UCP2*, *ESCO1*, and *TRPV6* marked peripheral nerve as an enriched (FDR-corrected *p* < 0.01) term, which agrees with the hypothesis that BBB is altered and brain-related genes can be picked up in blood. The most significant term (*p* < 0.01) for the Reactome pathway database was the immune system and was driven by genes including *NLRP1*, *LRR1*, *TMEM30A*, *DYNLL2*, *HERC4*, *IL2RB*, *ZBTB16*, and *SH2D1B*. The enriched term agreed with previous findings supporting that the immune system is disrupted in ASD patients. A full list of terms can be found in Supplementary Table S2. To evaluate functional profiling for specific subtypes, we selected top co-predictors (cf. section Materials and Methods) and visualized a fraction of functional genes in a heat map (Figure 6E). The analysis revealed that autism is again clearly distinguishable among other subtypes (Figure 6E) and was marked by membrane/transmembrane, metal ion binding, calcium channel activity, and ion transport. Furthermore, milder subtypes of ASD and control clustered together. Accordingly, PDD-NOS and AS were clustered together (Figure 6E) and annotated by terms related to the immune system, zinc ion binding, and nitrogen metabolic process. We also observed a high fraction of genes related to the peripheral nerve for all ASD subtypes (Figure 6E).

## Discussion

This study performed an IML analysis on multiple cohorts of control-case studies of ASD. Using the rule-based approach, we detected gene co-predictors that allowed to estimate dissimilarities between ASD subtypes and control. In total, we found 50 genes that were strong ASD predictors and were significantly enriched for functional pathways including the peripheral nerve and immune system. Results suggested that autism is the most severe form of ASD, while PDD-NOS is milder than autism and AS is the mildest form of ASD subtypes. Additionally, we found that PDD-NOS and AS are the most similar ASD subtypes. Furthermore, our analysis revealed a strong co-prediction mechanism between *EMC4* and *TMEM30* in the blood of ASD subjects.

Biomarkers that were detected in this analysis showed a satisfactory co-predictive power and distinguished ASD subtypes. One of the co-predictive mechanisms was the interaction between *EMC4* and *TMEM30A*, which interestingly are primarily involved in phospholipid transportation (Chen et al., 2011; Lahiri et al., 2014). The results of functional profiling uncovered that *EMC4* and *TMEM30A* are also associated with the peripheral nerve. Discovering peripheral nerve-related genes in blood samples supports the statement that BBB is altered in ASD (Fiorentino et al., 2016; Kealy et al., 2018). Interestingly, two of the main hubs, *HERC4* and *TMEM30A*, were included in immune system pathways. These findings suggested that core ASD co-predictors are linked to responses of the immune system and its signal can be detected in blood, as it has been reported earlier (Enstrom et al., 2009; He et al., 2019). To examine nonlinear and linear associations, we compared the most highly ranked co-predictors to the co-expression profiles. In the case of *EMC4*-*TMEM30A* and *MIA3*-*TMEM30A*, a co-regulation was notable (Supplementary Figures S6, S7, S10A,B). For *LRR1*-*RLF* and *COQ4*-*RLF*, the co-expression was weak; however, local strong evidence of co-prediction may not be evident from linear co-expression analysis (Supplementary Figures S8, S9, S10C,D). To confirm that suggested co-predictors reflect true biological interactions, these shall be further tested experimentally. Moreover, we observed that these co-predictive mechanisms are supported with various ASD subtypes. However, autism was the most supportive subtype (Figure 4).

The rule including *EMC4* and *TMEM30A* was significant in the IML models for DS1 and DS2 (FDR-corrected *p* < 0.05). *TMEM30A* encodes one of the *β* subunits that forms heterodimer with P4-ATPases and takes part in the process termed as lipid flipping (Chen et al., 2011). This process, which generates and maintains the phospholipid asymmetry in membranes, plays pivotal roles in membrane stability, vesicle trafficking, cell polarity and migration, and cell signaling (Yang et al., 2018). As one of the heterodimer partners of P4-ATPases, *TMEM30A* is required for the P4-ATPases to exit the endoplasmic reticulum (ER) and undergo transit to specific subcellular locations (Yang et al., 2018). *TMEM30A* has recently been demonstrated to play an essential role in the central nervous system (Yang et al., 2018). *TMEM30A* deficiency in the cerebellum results in protein folding and transport defects, which further induced ER stress response and apoptotic cell death. *EMC4* encodes a subunit of the conserved ER membrane protein complex (*EMC*), which is involved in phospholipid synthesis in the ER and in transfer-synthesized phospholipid from the ER to mitochondria (Lahiri et al., 2014). Recently, *EMC* has been proved to be a transmembrane domain insertase that inserts various proteins into membranes (Guna et al., 2018). The various protein substrates that failed insertion properly due to malfunctioning *EMC* probably contribute to many of *EMC*’s reported phenotypes, such as ER stress, aberrant membrane protein trafficking or degradation, and altered lipid homeostasis (Guna et al., 2018). All these *EMC*-related phenotypes have also been proved to be tightly related to proper function of the nervous system and contributed to ASD (Tamiji and Crawford, 2010; Kitagishi et al., 2015; Kawada and Mimori, 2018). The observed increased expression of *TMEM30A* together with the decreased expression of *EMC4* in ASD patients might contribute to the morphology changes of the cell membrane in the red blood cells of ASD subjects (Giacometti et al., 2017). Whether *TMEM30A* and *EMC4* could be further utilized in molecular diagnosing of ASD patients warrants further investigation.

Machine learning can provide novel insights into medical and biological questions, but it is not a panacea (Rajkomar et al., 2019; Roscher et al., 2020). In this study, we focused on patterns that describe potential molecular mechanisms for ASD subtypes, rather than only on the estimation of high-quality models. Thus, in order to perform high-quality learning, we encourage employment of other techniques such as deep learning (LeCun et al., 2015). To generate highly accurate IML models, we paid special attention to data preprocessing and important classification aspects such as removing batch effects and balancing the data, removing feature selection bias, adjusting FRs thresholds, and using CV for feature selection and classification. To test our methodology, we used three datasets. However, the pipeline is not limited by the number of datasets and it would be interesting to add more transcriptomics datasets. The methodology can be used with any omics-based data that can be represented as a decision table. For example, analysis on multiple DNA methylation datasets could be also performed. Moreover, the pipeline is flexible so it can be executed using other feature selection methods and rule-based algorithms. Furthermore, recent studies have highlighted the importance of using machine learning algorithms for multi-omics data analysis (Lin and Lane, 2017; Nicora et al., 2020). Herein, we demonstrated that the legibility of rule-based models can be utilized for integrative analysis of single-type omics data. Thus, the results of our analysis established the backbone for designing a multi-omics pipeline in the future.

There are some limitations in this study. In IML, the continuous space of the data is converted into a discrete space; thus, some information is lost. However, converting the data into a discrete space is a crucial step for rough set-based modeling. On the other hand, the discrete character of the data may prevent outliers from introducing bias to the analysis. Another limitation is that our analysis focused on existing ASD subgroups. In recent years, various studies (Ali et al., 2018; Orange et al., 2018; Gao et al., 2019; Matsui et al., 2020; Sinkala et al., 2020) aimed at finding novel disease subtypes that were characterized with specific molecular patterns. Thus, it would be interesting to modify the pipeline to allow identification of novel clinical subgroups, which would be especially interesting for PDD-NOS which is a subtype of unspecified ASD cases.

Our study aims at finding local and supervised co-regulation mechanisms; therefore, it is hardly comparable with co-expression mechanisms that frequently work in unsupervised and global way. In contrast, the concept of co-expression networks, for example, weighted correlation network analysis (Langfelder and Horvath, 2008), differs largely from the co-predictive networks in various aspects. The main difference is that co-predictive networks estimate dependencies in a supervised way, i.e., for a specific group of subjects in a given decision class. Thus, the rule-based approach reveals local dependencies. Unlike the rule-based approach, co-expression algorithms investigate global dependencies in an unsupervised manner (Butte and Kohane, 1999). Another aspect is that the co-expression approach investigates all genes to find dependencies and then the threshold is estimated to select the most co-expressed genes from the network. In a co-predictive approach, only the most relevant genes are investigated and their contribution to the supervised learning process is estimated. Thus, the interpretability of co-predictive networks is specified by the IML model. Additionally, a single rule can detect two or more dependencies and its statistics are based on supervised measurements. Ultimately, co-predictive and co-expression networks may both suggest co-regulation; however, their definitions are not interchangeable and their statistics are not comparable.

In summary, we showed that rule-based IML is a powerful technique for merging datasets and estimating dissimilarities between clinical subgroups. Our findings proved that IML rule-based modeling is a powerful method for integrating datasets, finding significant co-predictive mechanisms and revealing dissimilarities between clinical subgroups. To our knowledge, no other studies have performed IML modeling with the rule-based approach for merging the omics data and applied co-predictive networks for estimating subgroups dissimilarities. Thus, we believe that our methodology and results shed light for a novel approach of interpreting classification mechanisms for bioinformatics analyses. We hope that our pipeline will support clinicians and researchers for better diagnosis and analysis of ASD and other inherited disorders in the future.

## Data Availability Statement

Publicly available datasets were analyzed in this study. This data can be found at Gene Expression Omnibus: GSE18123 (https://www.ncbi.nlm.nih.gov/geo/query/acc.cgi?acc=GSE18123), GSE25507 (https://www.ncbi.nlm.nih.gov/geo/query/acc.cgi?acc=GSE25507) and GSE6575 (https://www.ncbi.nlm.nih.gov/geo/query/acc.cgi?acc=GSE6575).

## Author Contributions

MG and KS contributed conception, data collection of the study, and wrote the first draft of the manuscript. MG, KS, KD, KM, LF, and JK contributed design of the study. MG performed the data analyses. GP and KD wrote sections of the manuscript. All authors contributed to critical revision and approved the final version of the manuscript for publication.

### Conflict of Interest

The authors declare that the research was conducted in the absence of any commercial or financial relationships that could be construed as a potential conflict of interest.
