# Supplementary material for: Interpretable Machine Learning Reveals Dissimilarities Between Subtypes of Autism Spectrum Disorder
Source: Front Genet. 2021 Feb 25;12:618277. doi: 10.3389/fgene.2021.618277 (PMC7946989; doi:10.3389/fgene.2021.618277)
Supplement: Supplementary file 2 [file Data_Sheet_1.docx]

*Supplementary Material*

**Supplementary Figures**


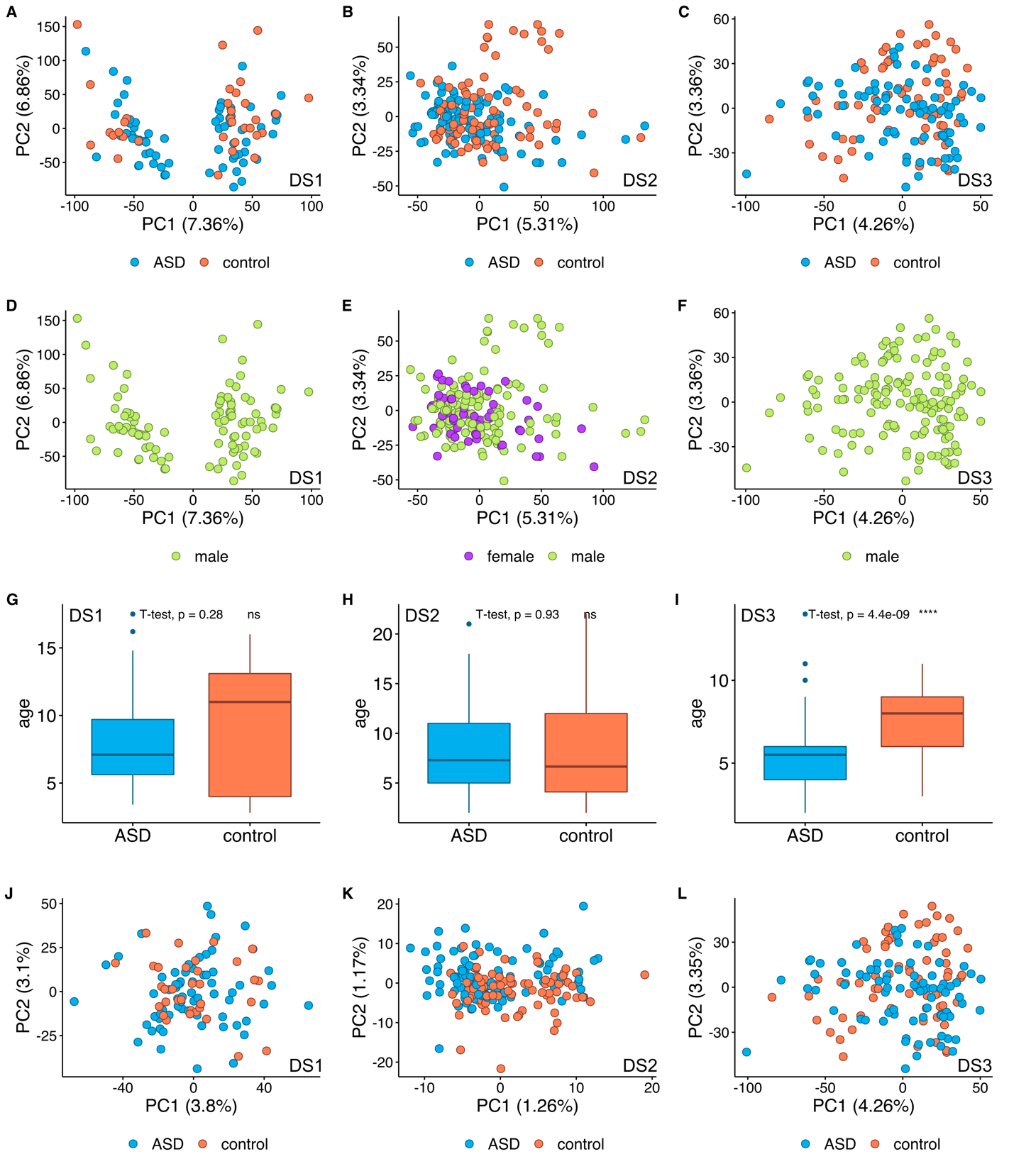


**Supplementary Figure 1**. Evaluation of known and unknown sources of batch effects for DS1-DS3. (**A-C**) Principal Component Analysis (PCA) performed on transcriptomics datasets before batch effect correction. Samples were colored according to decision class. (**D-F**) PCA performed on transcriptomics datasets before batch effect correction. Samples were colored according to sex. (**G-I**) Distribution of the age of subjects (in years) across control and autism spectrum disorder (ASD). Values of *p* from Student's t-test were marked as: ns(*p* > 0.05), *(*p* ≤ 0.05), **(*p* ≤ 0.01), ***(*p* ≤ 0.001) and ****(*p* ≤ 0. 0001). (**J-L**) PCA performed on transcriptomics datasets after batch effect correction. Samples were colored according to decision class.


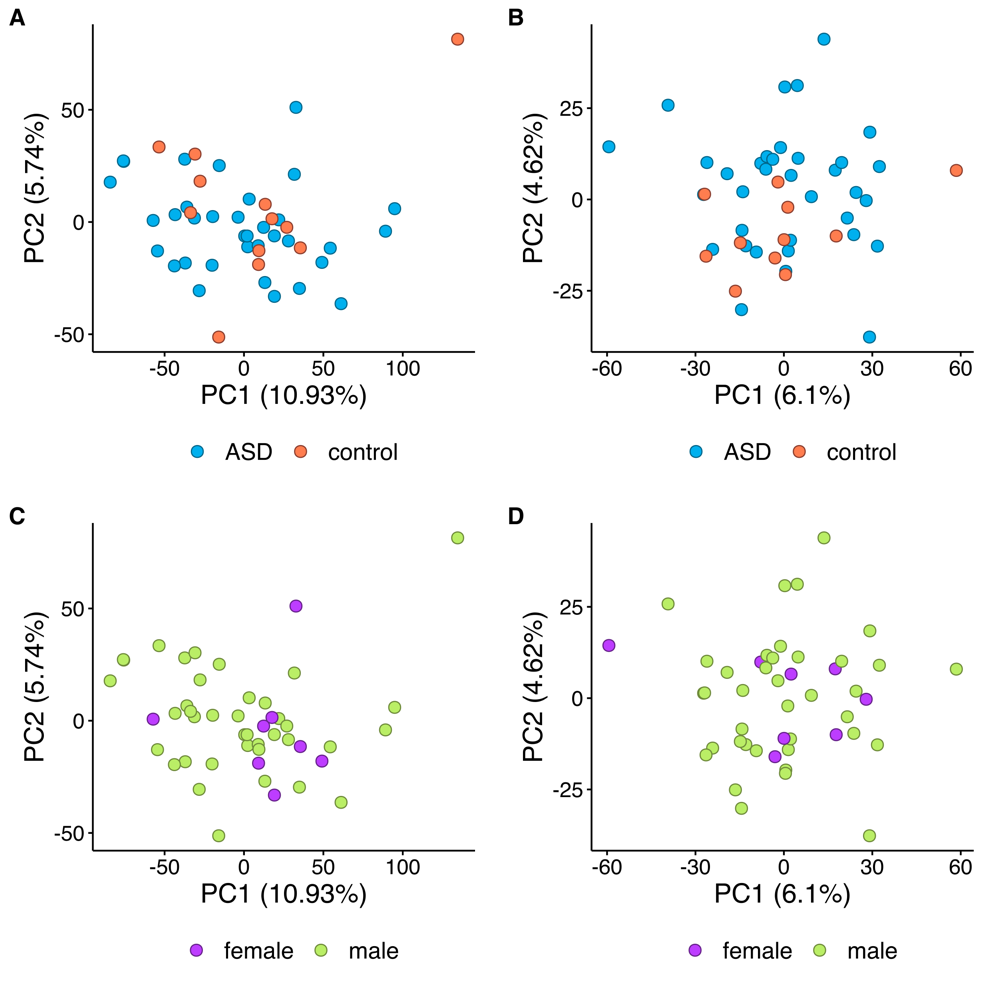


**Supplementary Figure 2**. Evaluation of known and unknown sources of batch effects for DS4. PCA performed before (**A,C**) and after (**B,D**) batch effect correction. Samples were colored according to decision class (**A,B**) and sex (**C,D**). Point in the upper right corner (male control sample; **A,C**) was treated as an outlier and removed (**B,D**).


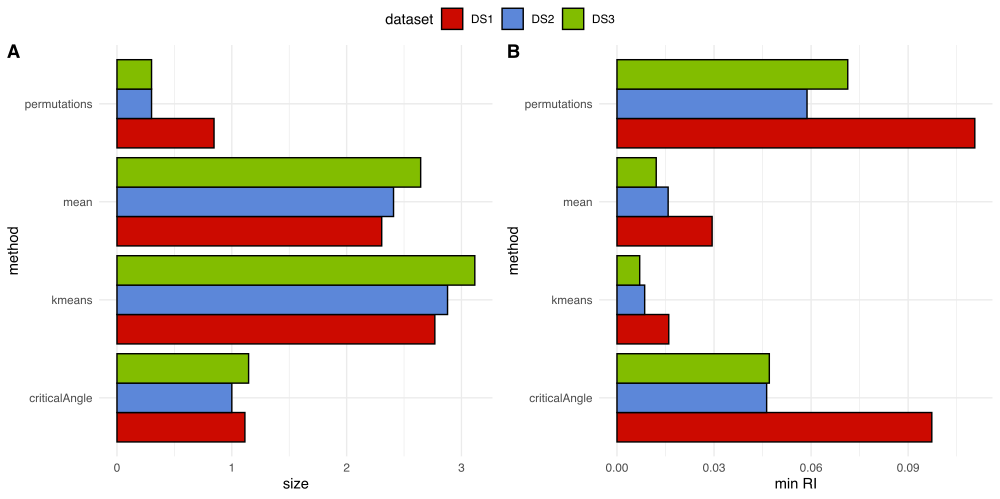


**Supplementary Figure 3.** Thresholds for selecting number of features estimated with four methods from an R package for Monte Carlo feature selection. (**A**) Size that is defined as the number of top features to select. Values are presented on a base-10 log scale. (**B**) Cutoff to select minimum relative importance (RI).


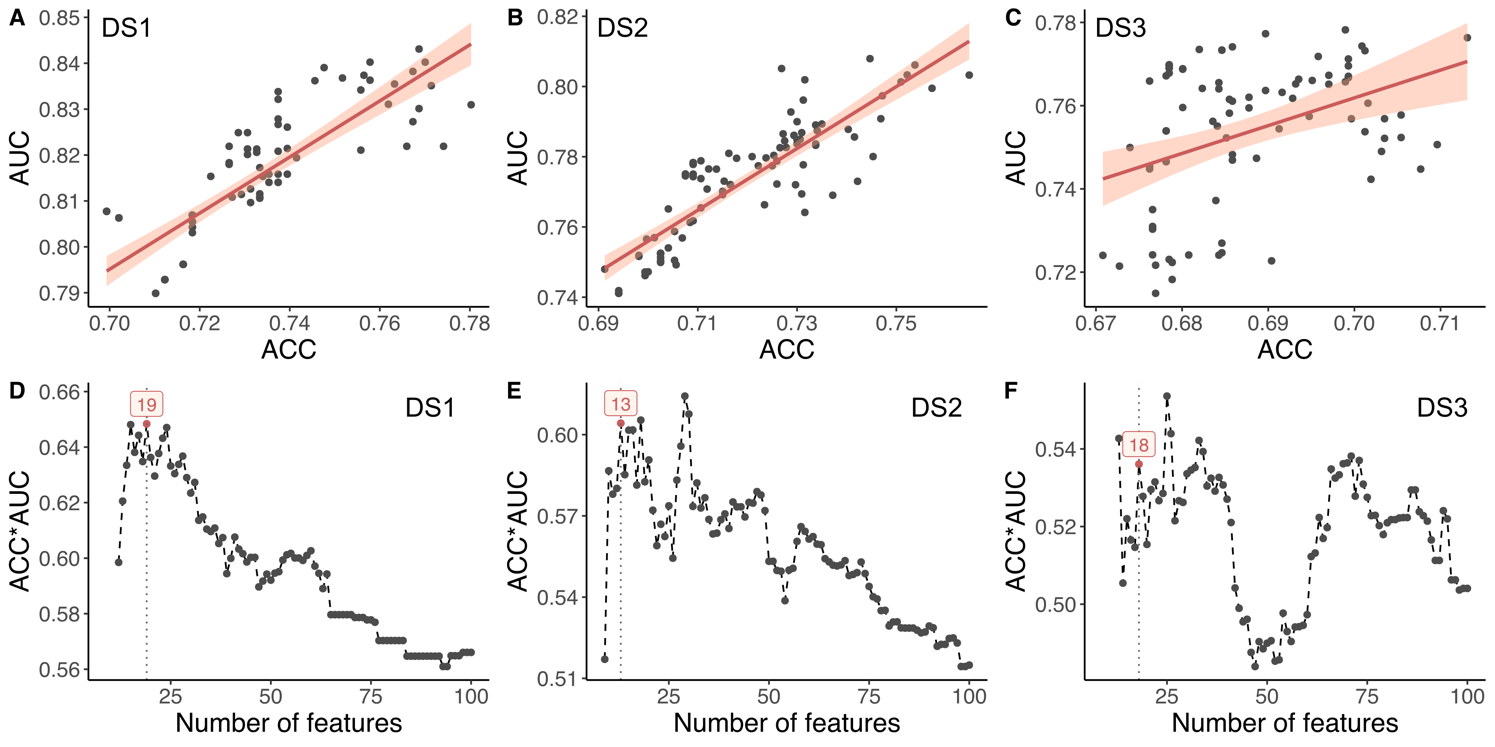


**Supplementary Figure 4.** Visualization of the procedure of adjusting feature rankings. (**A-C**) Model quality is shown as a function of area under the ROC curve (AUC) vs accuracy (ACC) for a particular dataset. Linear regression was applied and marked as a red solid line. (**D-F**) Estimation of the adjusted number of features based on the first local maximum (dotted line) of model quality. Local maximum is taken for the window size equal to 9. The adjusted number of features is given in the red boxes.


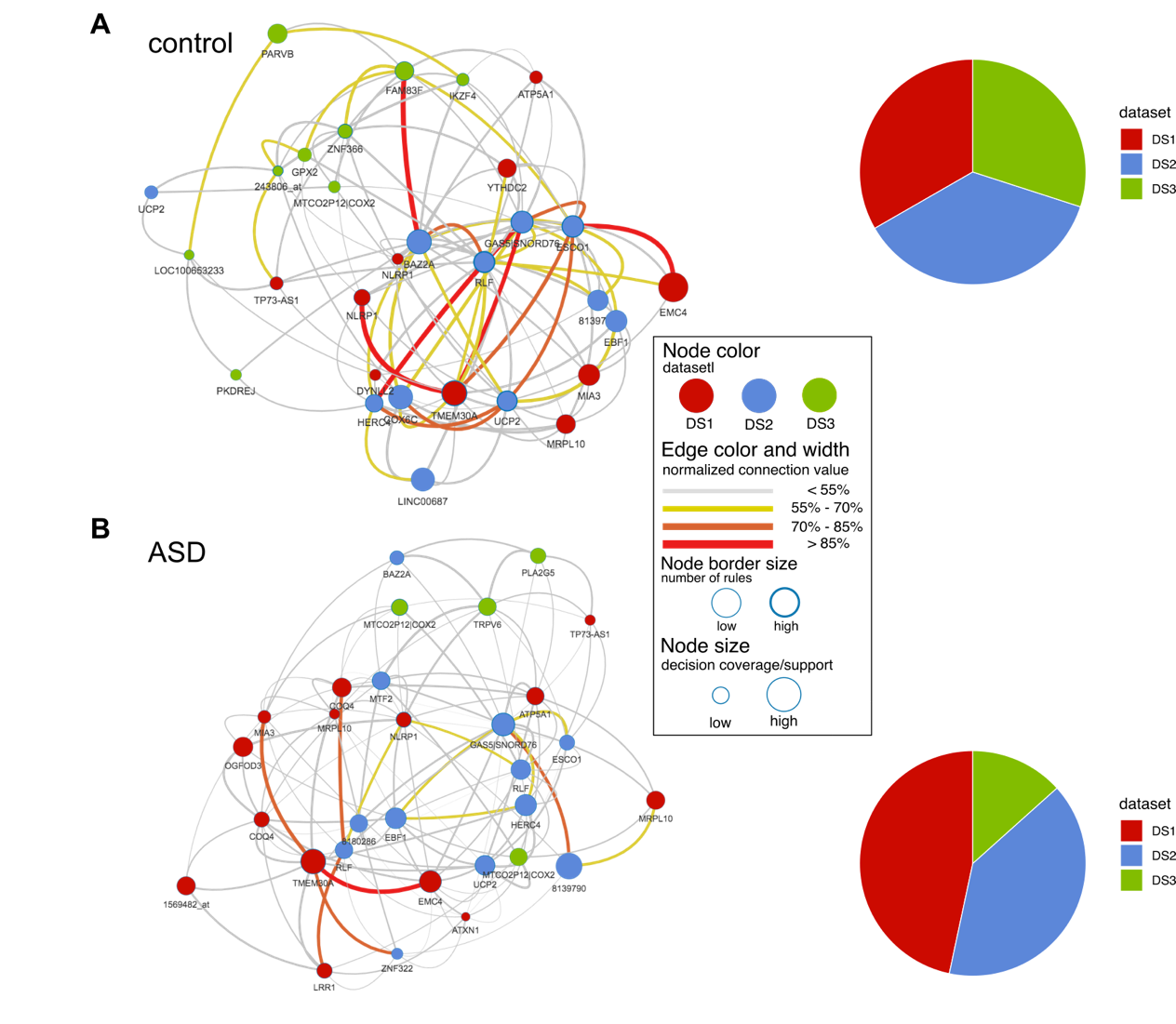


**Supplementary Figure 5.** Rule-based networks displaying contribution of features across datasets to IML modeling. (**A**) Network displays top 30 nodes from the rules corresponding to the control class. Nodes were selected for coverage above 0.05. Pie chart displays the distribution of nodes between datasets of the network made for the control class. (**B**) Network displays top 30 nodes from the rules corresponding to the ASD class. Nodes were selected for coverage above 0.05. Pie chart displays the distribution of nodes between datasets of the network made for the ASD class.

**
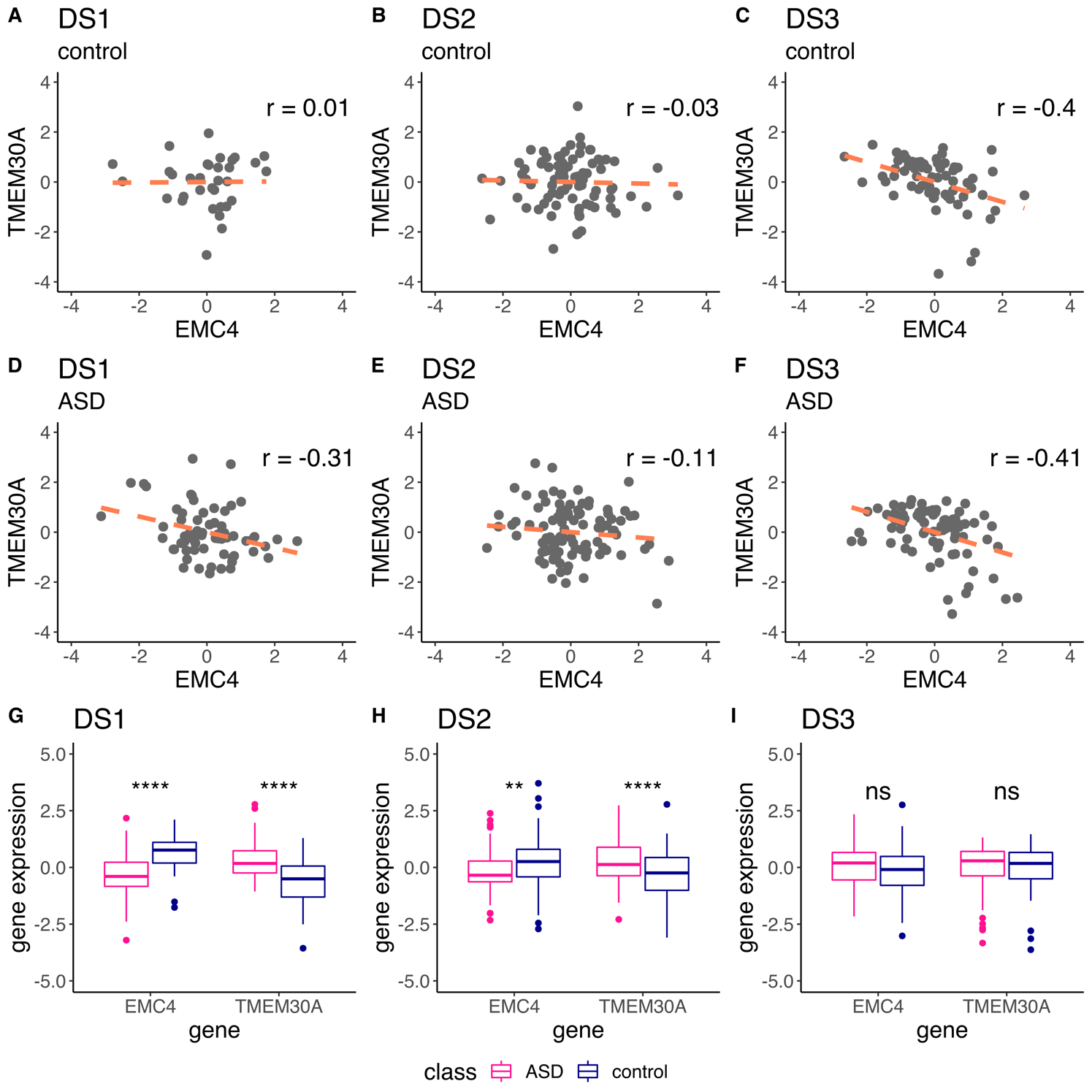
**

**Supplementary Figure 6.** Co-regulation mechanisms of *EMC4* and *TMEM30A* for control and ASD samples. Gene expression values were scaled around 0. Dotted orange line indicates a fitted linear model. (**A-F**) Pearson correlation between genes for a particular decision class. The correlation coefficient is given as *r*. (**G-I**) Gene expression changes between ASD and control for a particular gene. Thresholds for values of *p* were marked as follows: ns (*p* > 0.05), *(*p* ≤ 0.05), **(*p* ≤ 0.01), ***(*p* ≤ 0.001) and ****(*p* ≤ 0. 0001).


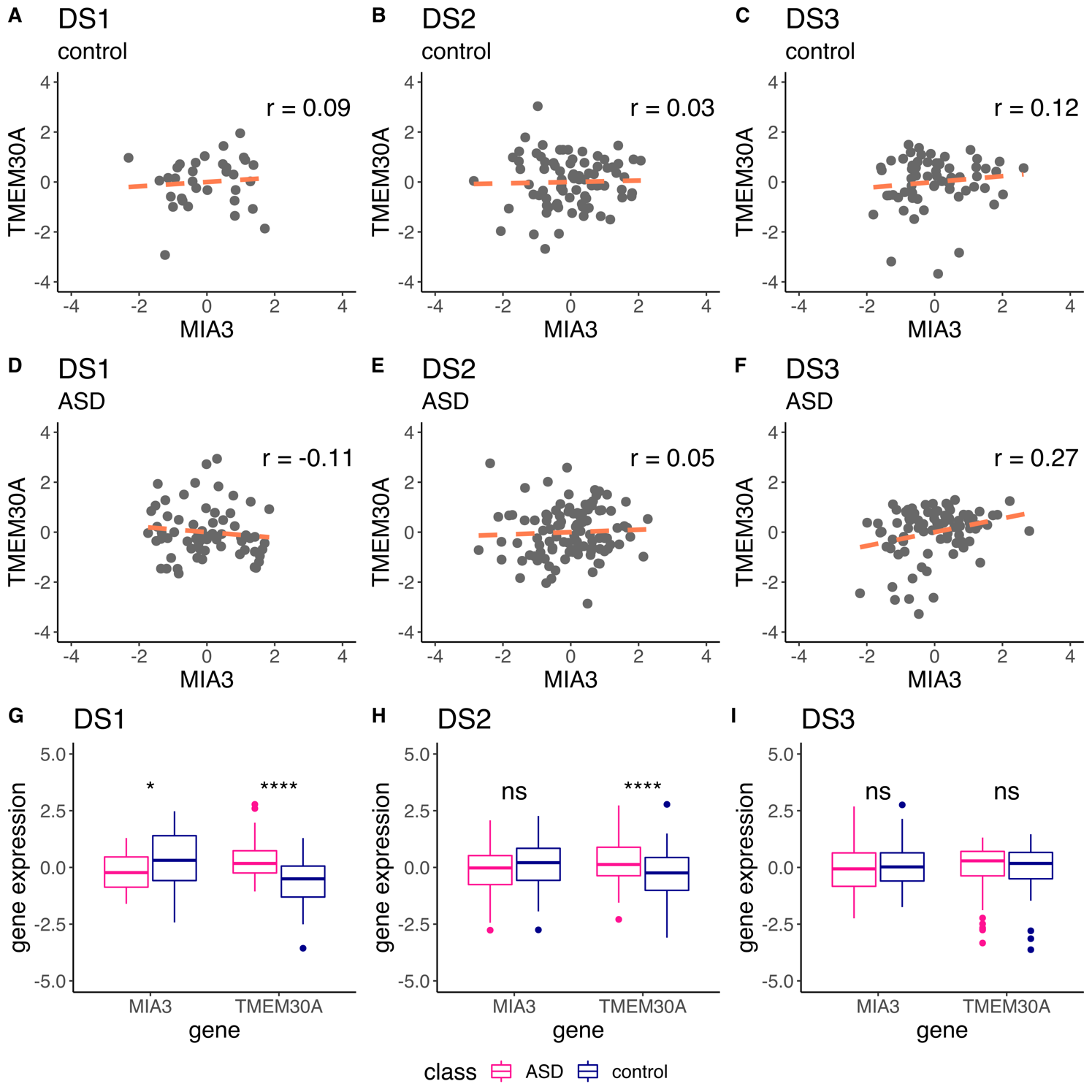


**Supplementary Figure 7.** Co-regulation mechanisms of *MIA3* and *TMEM30A* for control and ASD samples. Gene expression values were scaled around 0. Dotted orange line indicates a fitted linear model. (**A-F**) Pearson correlation between genes for a particular decision class. The correlation coefficient is given as *r*. (**G-I**) Gene expression changes between ASD and control for a particular gene. Thresholds for values of *p* were marked as follows: ns (*p* > 0.05), *(*p* ≤ 0.05), **(*p* ≤ 0.01), ***(*p* ≤ 0.001) and ****(*p* ≤ 0. 0001).


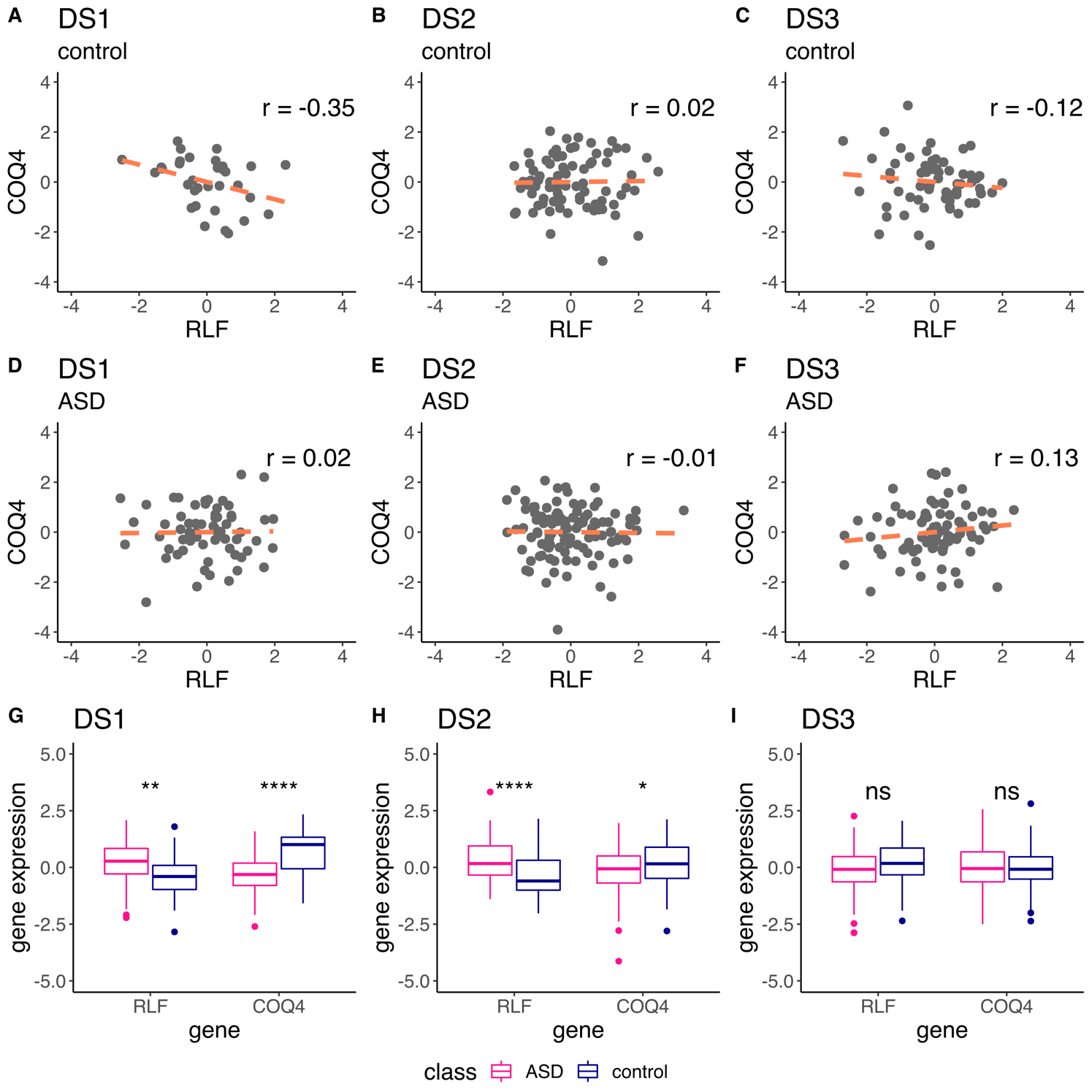


**Supplementary Figure 8.** Co-regulation mechanisms of *COQ4* and *RLF* for control and ASD samples. Gene expression values were scaled around 0. Dotted orange line indicates a fitted linear model. (**A-F**) Pearson correlation between genes for a particular decision class. The correlation coefficient is given as *r*. (**G-I**) Gene expression changes between ASD and control for a particular gene. Thresholds for values of *p* were marked as follows: ns (*p* > 0.05), *(*p* ≤ 0.05), **(*p* ≤ 0.01), ***(*p* ≤ 0.001) and ****(*p* ≤ 0. 0001).


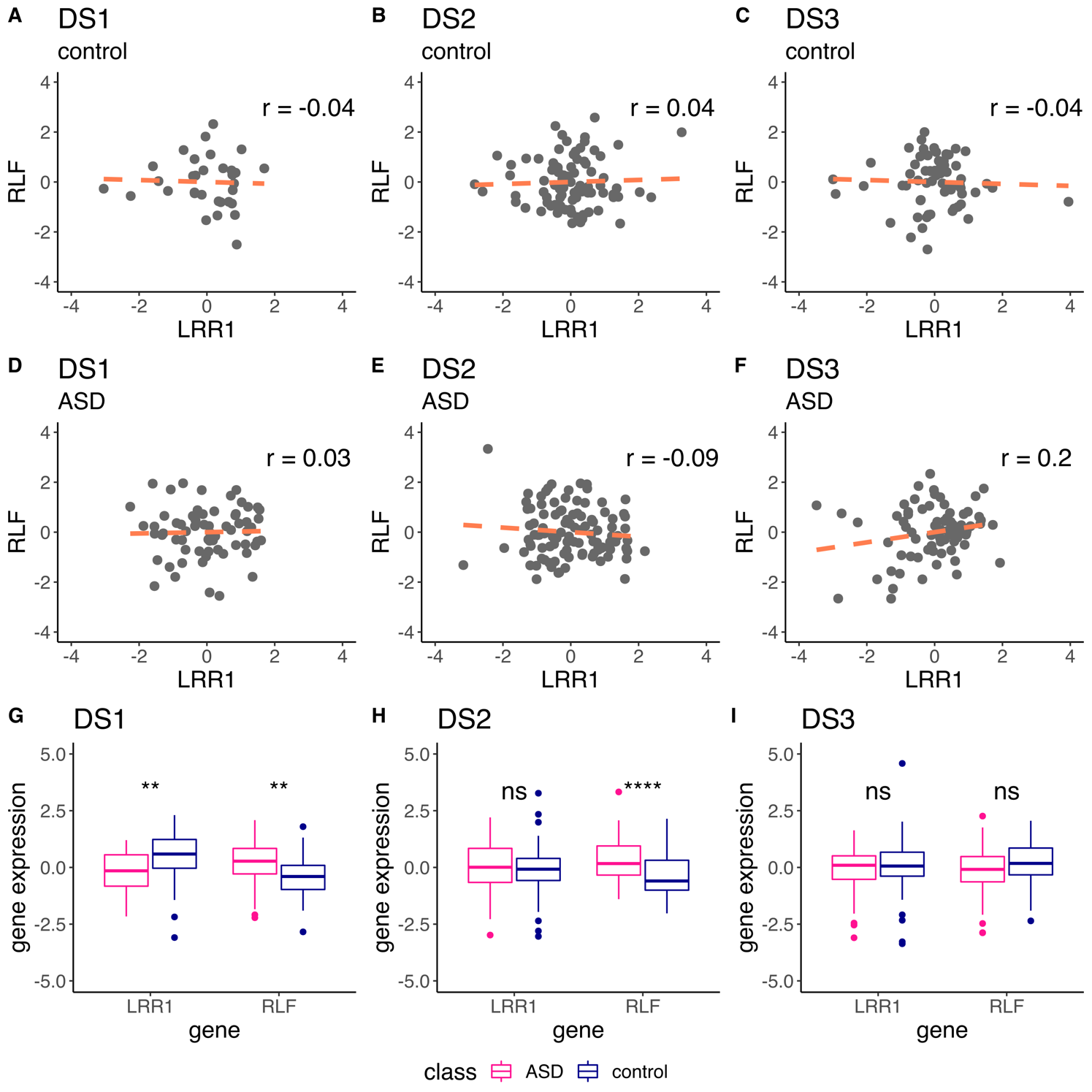


**Supplementary Figure 9.** Co-regulation mechanisms of *LRR1* and *RLF* for control and ASD samples. Gene expression values were scaled around 0. Dotted orange line indicates a fitted linear model. (**A-F**) Pearson correlation between genes for a particular decision class. The correlation coefficient is given as *r*. (**G-I**) Gene expression changes between ASD and control for a particular gene. Thresholds for values of *p* were marked as follows: ns (*p* > 0.05), *(*p* ≤ 0.05), **(*p* ≤ 0.01), ***(*p* ≤ 0.001) and ****(*p* ≤ 0. 0001).


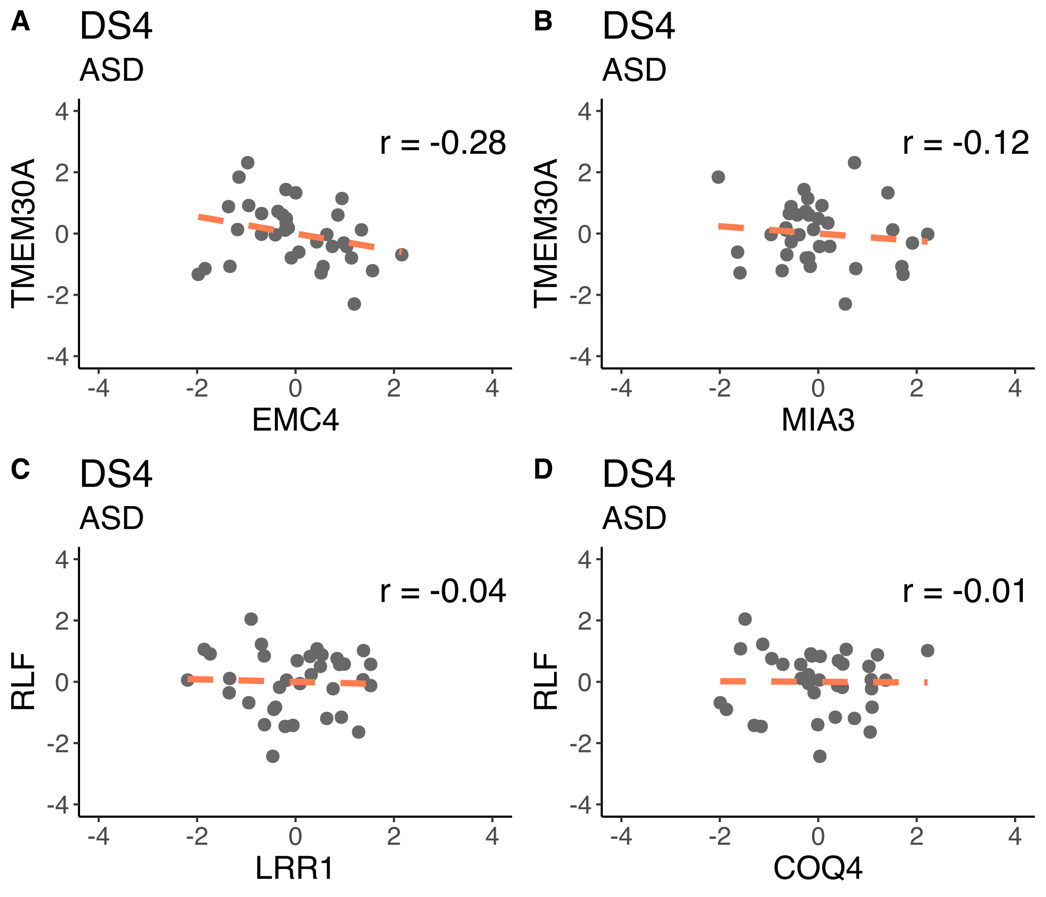


**Supplementary Figure 10.** Co-regulation mechanisms of the strongest co-predictive genes for ASD samples in DS4. Gene expression values were scaled around 0. Dotted orange line indicates a fitted linear model. Pearson correlation between genes for a particular decision class. The correlation coefficient is given as *r*.


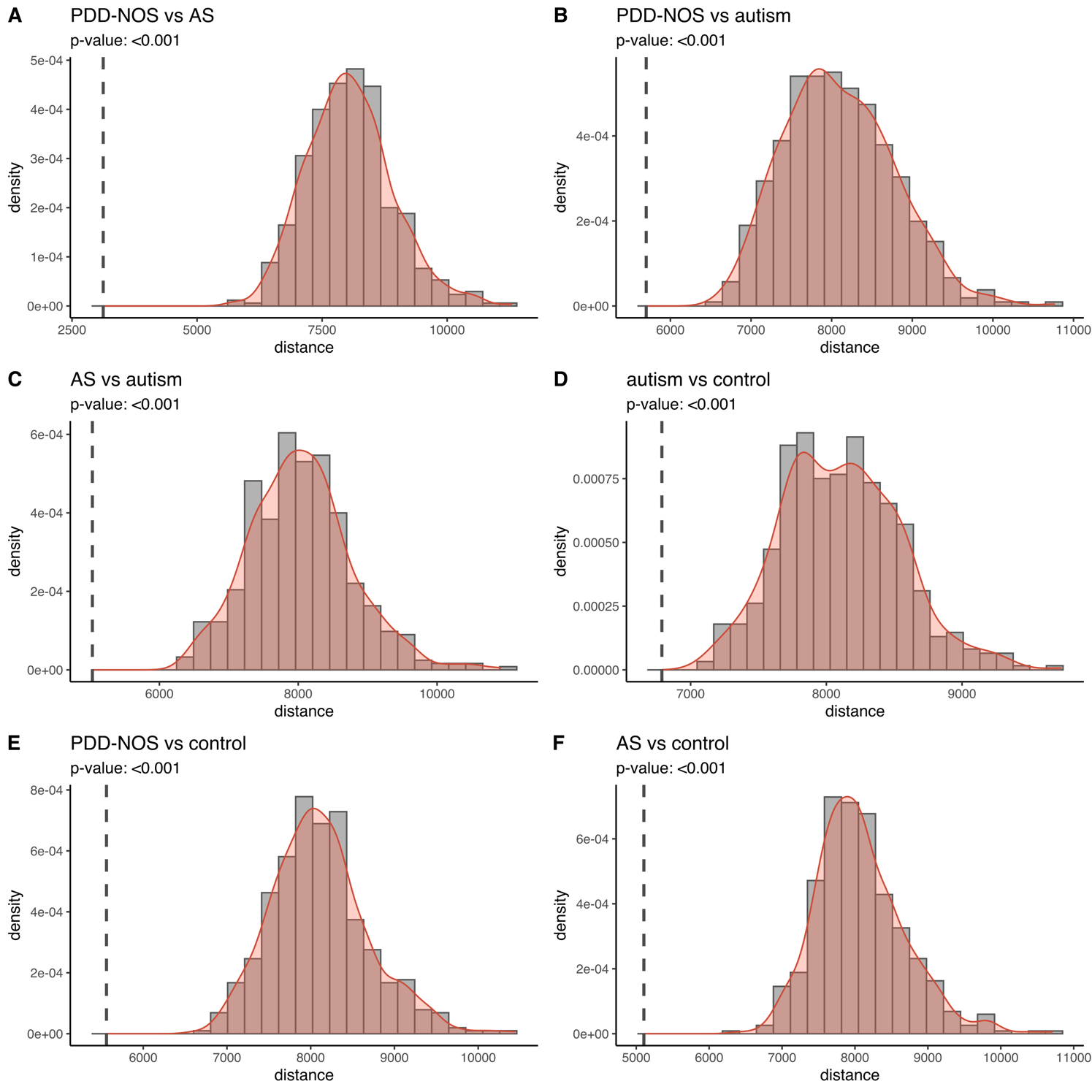


**Supplementary Figure 11.** Results of permutation tests from dissimilarity estimation for ASD subtypes and control. Distance values were estimated as the betweenness centrality distance in-between topological structures of networks. Permutations were executed on each pair of balanced networks. Left-tailed value of *p* was printed in the figures. A dashed line marks the non-permuted distance between subnetworks.


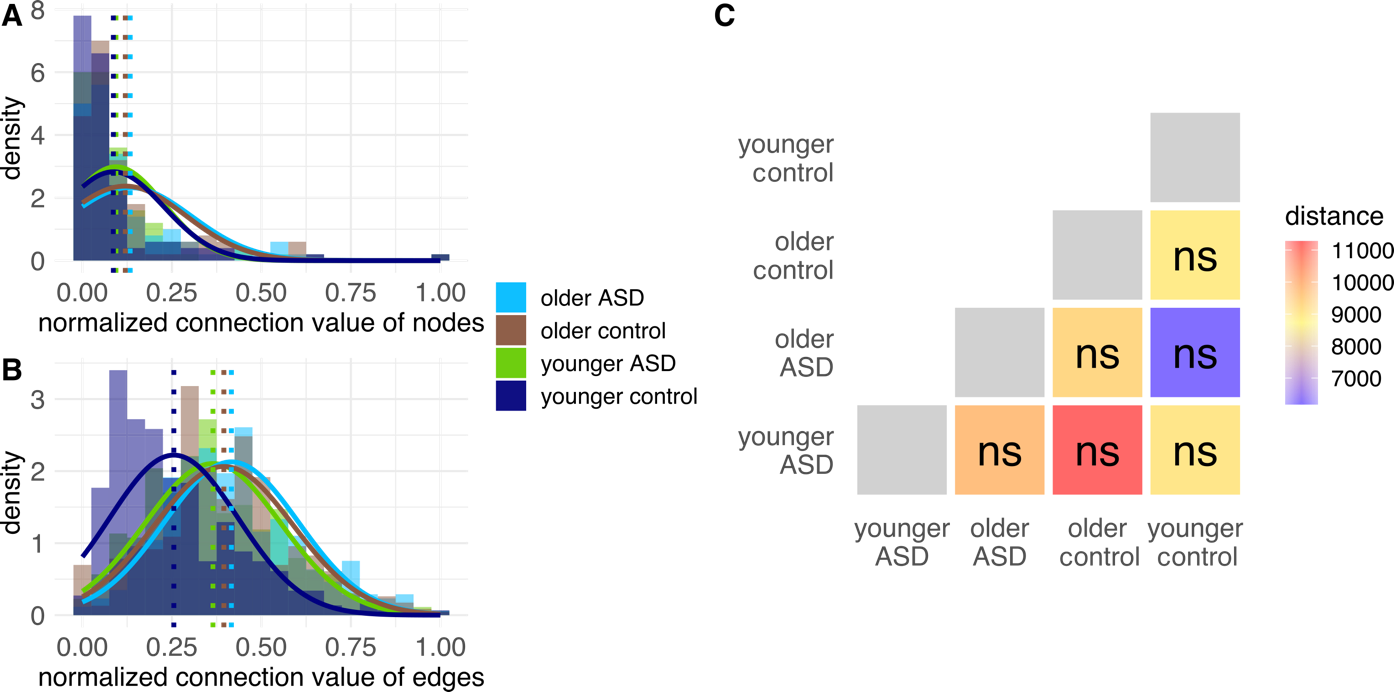


**Supplementary Figure 12.** Histogram of connection values for (**A**) nodes and (**B**) edges. Each histogram represents subnetwork that correspond to the particular age subgroup. Modeled distributions were marked for each subgroup. Dotted lines represent the average values for each distribution. (**C**) In-between-networks dissimilarity estimated as a centrality betweenness distance. Values of *p* were marked as ns (*p* > 0.05), *(*p* ≤ 0.05), **(*p* ≤ 0.01) and ***(*p* ≤ 0.001).

**
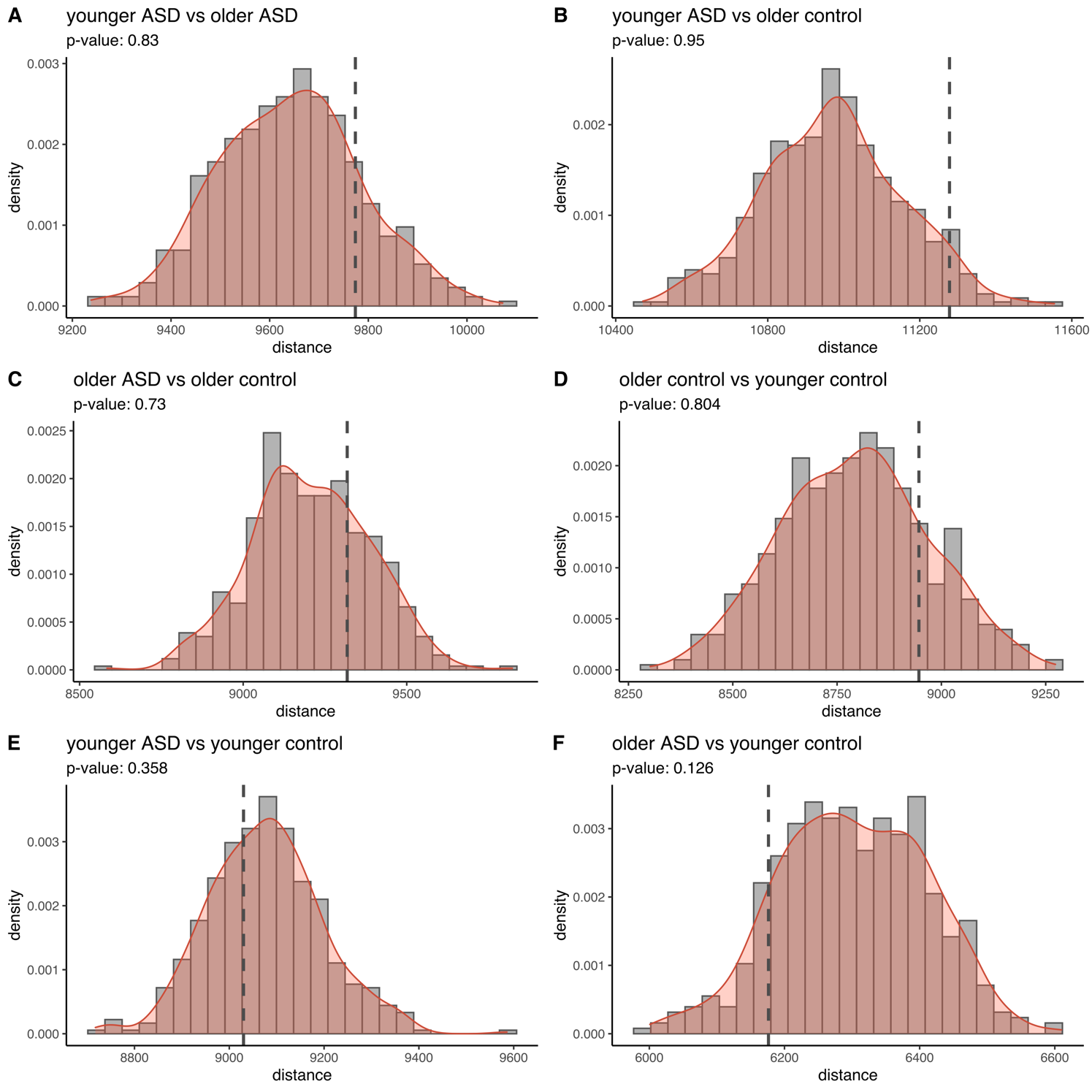
**

**Supplementary Figure 13.** Results of permutation tests from dissimilarity estimation for age subgroups. Distance values were estimated as the betweenness centrality distance in-between topological structures of networks. Permutations were executed on each pair of balanced networks. Left-tailed value of *p* was printed in the figures. A dashed line marks the non-permuted distance between subnetworks.


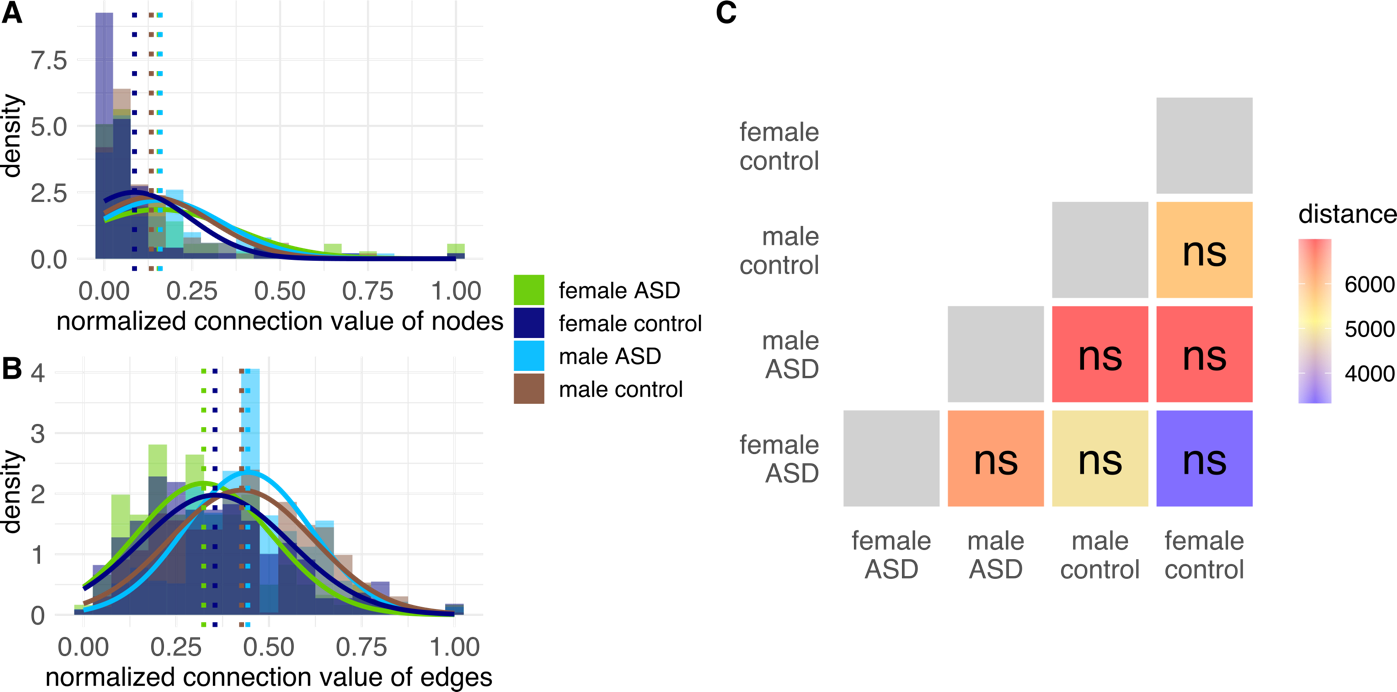


**Supplementary Figure 14.** Histogram of the connection values for (**A**) nodes and (**B**) edges. Each histogram represents subnetwork that correspond to the particular sex subgroup. Modeled distributions were marked for each subgroup. Dotted lines represent the average values for each distribution. (**C**) In-between-networks dissimilarity estimated as a centrality betweenness distance. Values of *p* were marked as ns (*p* > 0.05), *(*p* ≤ 0.05), **(*p* ≤ 0.01) and ***(*p* ≤ 0.001).


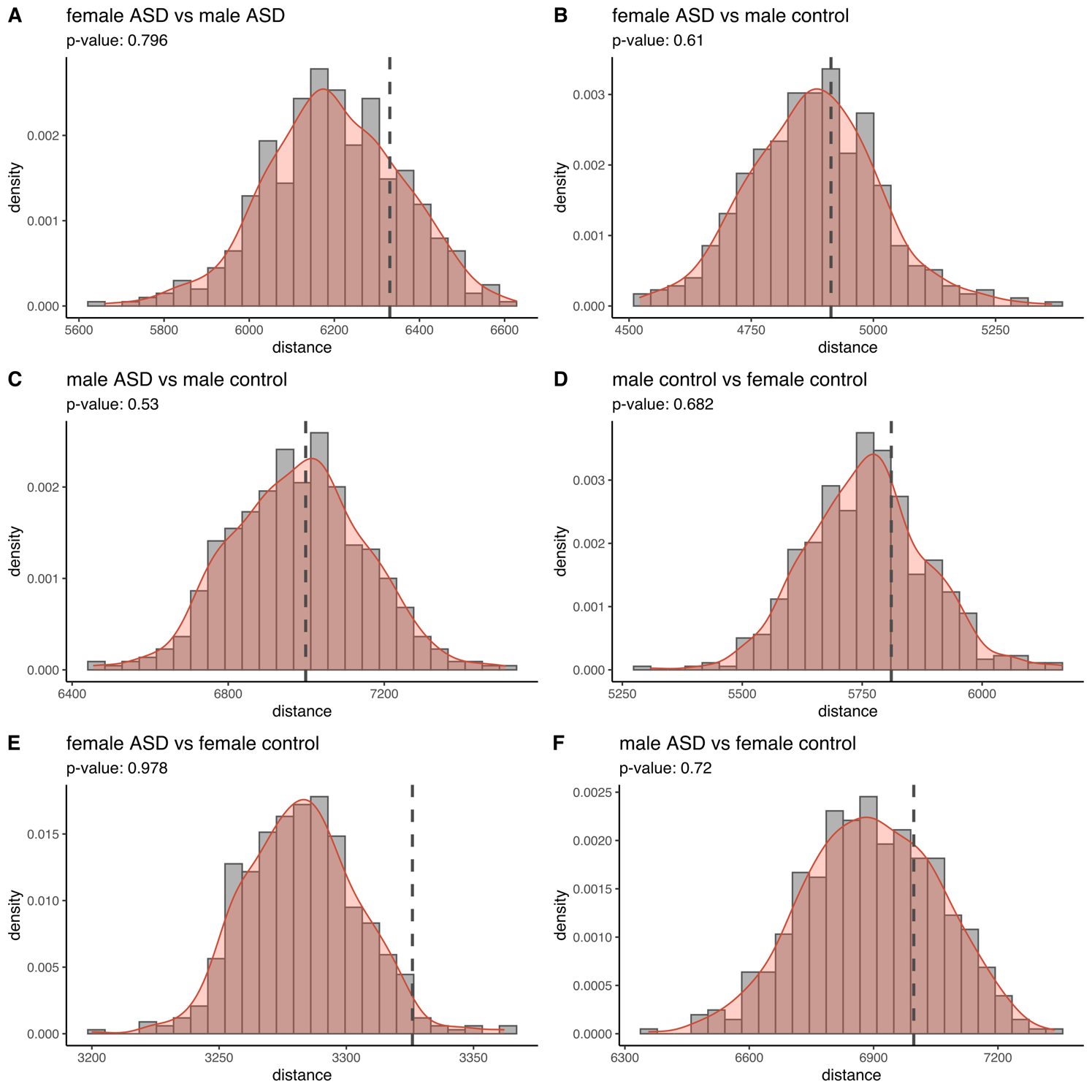


**Supplementary Figure 15.** Results of permutation tests from dissimilarity estimation for sex subgroups. Distance values were estimated as the betweenness centrality distance in-between topological structures of networks. Permutations were executed on each pair of balanced networks. Left-tailed value of *p* was printed in the figures. A dashed line marks the non-permuted distance between subnetworks.
